# The metabolic network coherence of human transcriptomes is associated with genetic variation at the cadherin 18 locus

**DOI:** 10.1007/s00439-019-01994-x

**Published:** 2019-03-09

**Authors:** Kristina Schlicht, Piotr Nyczka, Amke Caliebe, Sandra Freitag-Wolf, Annique Claringbould, Lude Franke, Urmo Võsa, BIOS Consortium, Sharon L. R. Kardia, Jennifer A. Smith, Wei Zhao, Christian Gieger, Annette Peters, Holger Prokisch, Konstantin Strauch, KORA Study Group, Hansjörg Baurecht, Stephan Weidinger, Philip Rosenstiel, Marc-Thorsten Hütt, Carolin Knecht, Silke Szymczak, Michael Krawczak

**Affiliations:** 1 Institute of Medical Informatics and Statistics, Kiel University, University Hospital Schleswig-Holstein, 24105 Kiel, Germany; 2 Department of Life Sciences and Chemistry, Jacobs University, 28759 Bremen, Germany; 3 Department of Genetics, University Medical Center Groningen, University of Groningen, 9700 RB Groningen, The Netherlands; 4 Department of Epidemiology, University of Michigan, Ann Arbor, MI 48109 USA; 5 Research Unit Molecular Epidemiology, Helmholtz-Zentrum München-German Research Center for Environmental Health, 85764 Neuherberg, Germany; 6 Institute of Epidemiology, Helmholtz-Zentrum München-German Research Center for Environmental Health, 85764 Neuherberg, Germany; 7 Institute of Human Genetics, Helmholtz-Zentrum München-German Research Center for Environmental Health, 85764 Neuherberg, Germany; 8 Institute of Genetic Epidemiology, Helmholtz-Zentrum München-German Research Center for Environmental Health, 85764 Neuherberg, Germany; 9 Chair of Genetic Epidemiology, Institute of Medical Informatics, Biometry and Epidemiology, Ludwig-Maximilians University, 81377 Munich, Germany; 10 Department of Dermatology, University Hospital Schleswig-Holstein, 24105 Kiel, Germany; 11 Institute of Epidemiology and Preventive Medicine, University Hospital Regensburg, 93053 Regensburg, Germany; 12 Institute of Clinical Molecular Biology, Kiel University, University Hospital Schleswig-Holstein, 24105 Kiel, Germany

## Abstract

Metabolic coherence (MC) is a network-based approach to dimensionality reduction that can be used, for example, to interpret the joint expression of genes linked to human metabolism. Computationally, the derivation of ‘transcriptomic’ MC involves mapping of an individual gene expression profile onto a gene-centric network derived beforehand from a metabolic network (currently Recon2), followed by the determination of the connectivity of a particular, profile-specific subnetwork. The biological significance of MC has been exemplified previously in the context of human inflammatory bowel disease, among others, but the genetic architecture of this quantitative cellular trait is still unclear. Therefore, we performed a genome-wide association study (GWAS) of MC in the 1000 Genomes/ GEUVADIS data (*n* = 457) and identified a solitary genome-wide significant association with single nucleotide polymorphisms (SNPs) in the intronic region of the cadherin 18 (*CDH18*) gene on chromosome 5 (lead SNP: rs11744487, *p* = 1.2 × 10^− 8^). Cadherin 18 is a transmembrane protein involved in human neural development and cell-to-cell signaling. Notably, genetic variation at the *CDH18* locus has been associated with metabolic syndrome-related traits before. Replication of our genome-wide significant GWAS result was successful in another population study from the Netherlands (BIOS, *n* = 2661; lead SNP), but failed in two additional studies (KORA, Germany, *n* = 711; GENOA, USA, *n* = 411). Besides sample size issues, we surmise that these discrepant findings may be attributable to technical differences. While 1000 Genomes/GEUVADIS and BIOS gene expression profiles were generated by RNA sequencing, the KORA and GENOA data were microarray-based. In addition to providing first evidence for a link between regional genetic variation and a metabolism-related characteristic of human transcriptomes, our findings highlight the benefit of adopting a systems biology-oriented approach to molecular data analysis.

**Electronic supplementary material:**

The online version of this article (10.1007/s00439-019-01994-x) contains supplementary material, which is available to authorized users.

## Introduction

Over the last 15 years, the development of high-throughput molecular technologies has greatly improved the scope and prospects of biological and biomedical research. At the same time, however, this newly acquired ability to characterize biological entities in their entirety and in great detail has led to an increased need for more efficient and more powerful approaches to data analysis. Omics technologies such as next generation DNA sequencing, in particular, generate large amounts of high-dimensional data per study subject that need to be processed and contextualized further to facilitate their biological interpretation.

One way to meet this challenge is dimensionality reduction, which means transforming the original data into data of much lower complexity but at the same time preserving as much as possible, or necessary, of the important information included in the original data. However, classical dimensionality reduction techniques such as principal component analysis and multi-dimensional scaling are ‘agnostic’ in the sense that they do not take the specificities of the data into account and, hence, hold a risk of losing critical information. To some extent, these shortcomings may be overcome by contextualizing the data with external knowledge. In biology, in particular, comprehensive systemic information is often available, for example, in the form of biological networks that formally represent complex biochemical processes. In fact, using this type of information to lift the curse of dimensionality, in our view, represents an essential aspect of systems biology.

**Fig. 1:**
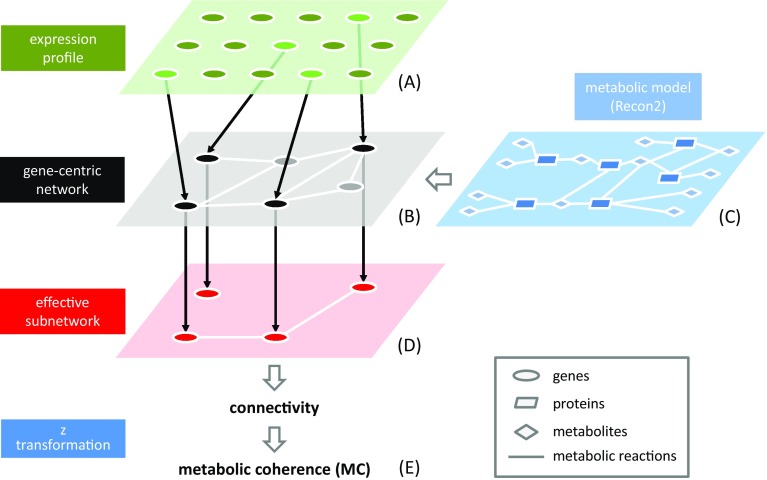
The principles of transcriptomic MC analysis. An individual expression profile is first dichotomized according to whether each gene is ‘saliently’ expressed or not (A). Then, the resulting binary signals are mapped onto a gene-centric network (B) that was derived earlier from a metabolic network (e.g., Recon 2) according to the functional correspondences between genes and proteins (C). Such mapping generates a profile specific, effective subnetwork of genes connected, or not, by reactions in the original metabolic network (D). Finally, the connectivity of the profile-specific subnetwork is calculated as the proportion of nodes connected to at least one other node (i.e., 3/4 in the present case), followed by a z transformation based on randomly chosen sets of genes (**e**)

Mapping experimental data onto a network allows the resulting network properties, including connectivity and vertex separation (Bonchev and Buck 2005), to be used as one-dimensional proxies of the original data. Along these lines, the concept of metabolic network coherence (MC) was introduced by Sonnenschein et al. (2011) to contextualize molecular data with a rich model of human metabolism (Fig. 1), currently Recon2 (Thiele et al. 2013). Since human diseases are often associated with metabolic disturbance, basing the dimensionality reduction of molecular data from a clinical context upon the Recon2 network may greatly facilitate a systems-orientated understanding of the biological processes involved in these conditions.

At the network level, Recon2 is a bipartite graph comprising metabolite and reaction nodes. Following Sonnenschein et al. (2011), Recon2 can be converted into a gene-centric network by consideration of the underlying gene-reaction associations. More specifically, two genes are connected by an edge in the resulting network if the reactions associated with these genes share a common metabolite (Fig. 1). To avoid implausibly short distances in the gene-centric network, however, the most highly connected metabolites (e.g., ATP and other ‘currency metabolites’) are removed from the original network prior to its projection onto the gene nodes, as suggested by Ma and Zeng (2003).

To calculate the MC of an individual-specific molecular dataset, the data are first dichotomized according to a particular gene-specific criterion, followed by mapping of the data onto the gene-centric network mentioned above (Fig. 1). The dichotomization process defines an individual-specific fragmentation of the gene-centric network when nodes (i.e., genes) exhibiting one of the two dichotomization states are eliminated, and the MC of the dataset is proportional to the connectivity of the resulting subnetwork (see “Materials and methods”). Calculation of MC thus transforms a high-dimensional molecular profile into a single quantitative trait that can be viewed as a phenotype of the respective individual, fit for further analysis such as, for example, classical statistical association tests.

In the case of transcriptome data, a natural choice of genes to be highlighted comprises those with significantly altered expression. This way, MC measures the extent to which the co-regulation of gene expression is explicable by the adjacency of the respective genes in the underlying network, where ‘adjacency’ means that gene products are involved in reactions sharing at least one metabolite. High MC can be interpreted as cells responding well to metabolic requirements in that gene products in simultaneous need are either both abundant or absent. Low MC, in contrast, means that the cells are less responsive and somehow ‘ignore’ these requirements.

Metabolic coherence was used successfully before to assist the interpretation of transcriptome data in the context of human disease. Drawing upon a study on pediatric inflammatory bowel disease, Knecht et al. (2016) demonstrated that the MC of intestinal transcriptomes manifests in two distinct types, with different statistical distributions, that occur at significantly different prevalence in patients and controls. Subsequently, Häsler et al. (2017) showed that transcriptomic MC is associated with disease-related changes in human mucosal tissue and microbiome.

So far, however, little is known about the genetic architecture of transcriptomic MC, i.e., of the number and identity of the genes that impact upon this trait. Therefore, we performed a genome-wide association study (GWAS) of transcriptomic MC in an attempt to map quantitative trait loci (QTL) that might not only explain the causes of natural variation in transcriptomic MC, but that may also contribute to a better understanding of the abovementioned disease associations. Worthy of note, in relating functional (i.e., network-based) information to genetic information, we follow Carter et al. (2013) who were among the first to view genetic mutations as network perturbations.

## Materials and methods

### Data characteristics and provenance

The present study drew upon transcriptome and genome data from four different human population-based studies (Table 1). In an exploratory, genome-wide association study (GWAS), we used RNA sequencing (RNA-seq) and SNP microarray data from the 1000 Genomes/GEUVADIS project (Lappalainen et al. 2013) comprising 457 samples from five different populations, namely Utah residents with Northern and Western European ancestry (CEU, *n* = 91), British (GBR, *n* = 93), Finns (FIN, *n* = 94), Tuscans (TSI, *n* = 91) and Yoruba (YRI, *n* = 88). A total of 241 donors (52.7%) were female.

**Table 1:** Description of study data (including cellular origin and molecular typing technology)

Acronym	1000 genomes/GEUVADIS	KORA	GENOA	BIOS
Design	Population-based	Population-based	Hypertensive index patients, siblings (affected and non-affected)	Population-based
Population	Mixed	German	US American of European descent (non-Hispanic)	Dutch
Sample size	457	711	411	2661
Cell type	Lympho-blastoid cell lines	Whole blood	Lympho-blastoid cell lines	Whole blood
Expression data	RNA-seq	Microarray	Microarray	RNA-seq
Genetic data	Microarray	Microarray	Microarray	Microarray

The replication of any significant GWAS findings was aimed at in three additional studies for which different types of transcriptome and genome data were available, namely (1) the German population-based KORA cohort F4 (Kooperative Gesundheitsforschung in der Region Augsburg; Holle et al. 2005; *n* = 711, 357 female), (2) US Americans of European, non-Hispanic descent from GENOA (Genetic Epidemiology Network of Arteriopathy; Daniels et al. 2004; *n* = 411, 233 female, 318 hypertensive) and (3) the Dutch BIOS consortium (Biobank-based Integrative Omics Study, *n* = 2661, 1480 female), also including data from the Genome of The Netherlands Consortium (Boomsma et al. 2014).

### Transcriptome data

1000 Genomes/GEUVADIS: Gene expression in lymphoblastoid cell lines (LCL) was quantified by RNA-seq on an Illumina HiSeq 2000 as described by Lappalainen et al. (2013). For the present study, expression data were downloaded from the GEUVADIS website (genome build hg19) in the form of RPKM values (Mortazavi et al. 2008), followed by log-transformation f(*x*) = log_2_(*x* + 1). Genes with raw sequencing counts of zero in > 50% of samples were removed, leaving 23,219 genes for subsequent analyses.

KORA: Gene expression in whole blood was quantified using the Illumina HT12 v3 microarray. Normalized and log-transformed expression data were provided by KORA for individuals with matching sex information. Only probes with ‘perfect’ or ‘good’ quality according to Barbosa-Morais et al. (2010), and with a detection *p* value < 0.05 in at least 50% of individuals, were considered further and mapped to Ensembl gene identifiers using Bioconductor package illuminaHumanv3.db. The resulting dataset comprised 9263 genes.

GENOA: Gene expression was measured in LCLs from 818 individuals (hypertension index patients, affected and non-affected siblings) using the Affymetrix Human Exon 1st microarray (Turner et al. 2009). Normalized data were downloaded from Gene Expression Omnibus (accession number GSE49531; Edgar et al. 2002). For consistency reasons, related individuals were excluded, keeping one randomly chosen individual per family for further analysis (*n* = 411; 230 affected, 181 non-affected). The final transcriptome dataset comprised 14,701 genes.

BIOS: Gene expression was quantified in whole blood by RNA-seq (Illumina HiSeq 2000) as described by Zhernakova et al. (2016). Transcriptome data were downloaded from the European Genome Phenome Archive (accession number EGAS00001001077) in accordance with relevant data access regulations. The data were normalized using the ‘Median Ratio Method’ as implemented in R package DESeq2, and genes with raw sequencing counts of zero in > 50% of samples were removed. A total of 21,616 genes were included for subsequent analyses.

### SNP genotype data

1000 Genomes/GEUVADIS: Genotypes used in the present study were generated on an Infinium Omni 2.5M microarray, targeting 2458,634 SNPs. Of the 462 samples for which both genotype and expression data were available, five were excluded because of inconsistent sex assignment. For the 457 remaining samples, SNPs were quality-controlled based on the following criteria: (1) call rate ≥ 0.99, (2) autosomal location, (3) minor allele frequency (MAF) > 0.05, (4) ≥ 5 samples homozygous for the minor allele and (5) Hardy–Weinberg equilibrium *p* ≥ 0.001 both in the YRI and in the European subgroups combined. This filtering step left 1067,702 SNPs for inclusion in the GWAS. SNP genotypes were encoded by minor allele dosage. After LD pruning, PCA of the SNP genotypes was carried out to evaluate population genetic differences between different populations using smartpca from the EIGENSOFT package (version 6.0.1; Patterson et al. 2006).

KORA: Genotypes imputed from the Affymetrix Human Axiom microarray using IMPUTE2 (Howie et al. 2012) were provided by KORA (Wichmann et al. 2005). Genotype probabilities were converted into minor allele dosage format. Some 272 SNPs with MAF ≥ 0.05 were found to be located in the 1 Mb region covering the human cadherin 18 (*CDH18*) gene and were analyzed in an attempt to replicate the main GWAS result.

GENOA: The GENOA participants were genotyped on three different SNP microarrays, namely Affymetrix AFFY 6.0, Illumina Human660W-Quad v1A and Illumina Human1M-Duov3 B. Quality control was carried out as described by Daniels et al. (2010). Dosage genotypes for the 262 SNPs with MAF ≥ 0.05 that were located in the *CDH18* region were provided to us by GENOA under a separate data sharing agreement.

BIOS: Samples in BIOS were also genotyped on several different SNP microarrays (for details, see Zhernakova et al. 2016) and the results were processed further using the Genotype Harmonizer (Deelen et al. 2014), followed by imputation using the Michigan imputation server (Das et al. 2016). Quality control was carried out as described by Zhernakova et al. (2016). Some 246 SNPs from the *CDH18* region (MAF ≥ 0.05) were included in the replication analysis.

### Metabolic network coherence (MC)

Transcriptomic metabolic network coherence (henceforth referred to as MC, for short) was calculated as described by Sonnenschein et al. (2011), with modifications subsequently proposed by the same group for human data (Sonnenschein et al. 2012). Since MC calculation requires binary input (Fig. 1), the gene expression data were dichotomized (‘normal’ vs ‘salient’) within each study according to whether or not a particular expression value belonged to the gene-specific upper or lower 2% quantile. Both tails of the distribution were considered simultaneously because our MC analysis was geared towards identifying gene pairs with pronounced co-regulation, i.e., comprised both concordant and discordant effects. In addition, adoption of the 2% quantile was not only found to be a meaningful choice in practice before (Knecht et al. 2016), but also represented a viable compromise between sensitivity and specificity with regard to the detection of salient gene expression.

The connectivity of the profile-specific subnetwork put up by the saliently expressed genes was determined by dividing the number of nodes of non-zero degree (i.e., nodes connected to at least one other node) by the total number of nodes of the subnetwork. The null distribution of the connectivity was obtained by simulation (*n* = 2000), each time drawing random gene sets from Recon2 that were of the same size as the subnetwork. The MC value of a gene expression profile was then defined as its z score with regard to the null distribution (Fig. 1). Further details on MC calculation can be found elsewhere (Knecht et al. 2016; Sonnenschein et al. 2011, 2012).

Calculation of the MC values required mapping of an expression profile onto the gene-centric network derived from human metabolic model Recon2 (Thiele et al. 2013). In the course of this, 5% so-called ‘currency metabolites’ (e.g., ATP) were excluded to avoid implausibly short distances in the ensuing gene-centric network, leaving 1660 Recon2 genes for further consideration. Of these, 1348 were present in the 1000 Genomes/ GEUVADIS transcriptome dataset, 896 occurred in KORA, 1302 in GENOA and 1358 in BIOS. Absent genes were treated as missing in subsequent analyses (i.e., the gene-centric networks were constructed without these genes). Here, ‘absent’ meant that the genes in question were either not covered by the respective typing technology at all or yielded expression values below the detection threshold in > 50% of samples, and hence did not exhibit sufficient variation for meaningful inclusion in the MC calculations. By far the strongest overlap in terms of the Recon2 genes present was observed between 1000 Genomes/GEUVADIS and BIOS (89% concordance). This came as no surprise because both datasets were generated by RNA sequencing, which is known to be a more sensitive and specific means of gene expression analysis than microarray-based typing. Consequently, the remaining concordance rates were found to be notably smaller, ranging from 56% for GENOA and KORA (different typing technology, different tissue) to 72% for 1000 Genomes/ GEUVADIS and GENOA (for further details, see Supplementary Figure S1 and Supplementary Table S1). In any case, the observed differences in gene content did not cause any notable inter-study differences in MC value distribution (Supplementary Figure S2).

Sensitivity analysis of the exploratory GWAS was carried out varying the percentage of excluded currency metabolites from 3 to 8% (in 1% steps) and of the threshold used to define salient gene expression from 1 to 3% (in 1% steps). Inter-population group differences in terms of quantitative variables were assessed for statistical significance using either a Wilcoxon rank sum test (2 groups) or a Kruskal–Wallis test (> 2 groups) as implemented in R v.3.4.3.

### Genome-wide association study (GWAS), replication and eQTL analysis

Linear models with MC as the continuous response variable and with the minor allele dosage genotype of an SNP as an influence variable were employed in the exploratory GWAS of the 1000 Genomes/GEUVADIS data. Each model included one SNP at a time and was adjusted for population affiliation and gender. Additional analyses adjusted for the top SNP as well were carried out to search for independent MC-genotype associations in the respective region. Functional annotation of the GWAS summary statistics was carried out on the FUMA platform (Watanabe et al. 2017) provided by the Complex Trait Genetics (CTG) laboratory at VU University Amsterdam, NL.

In our attempt to replicate the sole genome-wide significant GWAS signal in the other three studies, between 246 (BIOS) and 272 (KORA) SNPs located in the *CDH18* gene region (covering approximately 500 kb upstream and downstream of the top SNP) were included in linear models with MC as a response variable and the respective dosage genotype as an influence variable. The models were adjusted for gender (KORA), or gender and biobank source (BIOS), or gender, age and hypertension status (GENOA), respectively. To correct the significance level for multiple testing, the number of LD-effective SNPs in the *CDH18* gene region was determined using the Genetic Type 1 Error Calculator (Li et al. 2012).

A trans-eQTL analysis was performed in 1000 Genomes/ GEUVADIS to relate the expression values of the 1348 Recon2 genes to the genotypes of the SNPs from the *CDH18* gene region. *P* values from the respective Kruskal–Wallis tests were Bonferroni corrected, dividing the nominal significance threshold of 0.05 by the product of the number of Recon2 genes (*n* = 1348) and the number of LD-effective SNPs (*n* = 45; see Results).

In an attempt to resolve the MC association of the *CDH18* gene region further and to increase the statistical power of the eQTL analysis, we also performed hierarchical clustering of the dichotomized Recon2 gene expression values using R functions dist (method ‘binary’) and hclust (setting ‘ward.D’). The result was subsequently employed to divide the Recon2 genes into sub-clusters that efficiently reflected the outcome of the eQTL analysis. To this end, a threshold to the cluster height was gradually reduced until a Kruskal–Wallis test of the gene-specific minimum *p* values from the eQTL analysis indicated nominally significant heterogeneity between the emerging sub-clusters (Kruskal–Wallis *p* < 0.05). The sub-cluster with the smallest median of the gene-specific minimum *p* values was then subjected to biological theme enrichment analysis with DAVID 6.8 (Huang et al. 2009), using default settings. The analysis was run against the list of Recon2 genes as background, and only enriched terms comprising at least 10 genes were considered further.

## Results

**Fig. 2:**
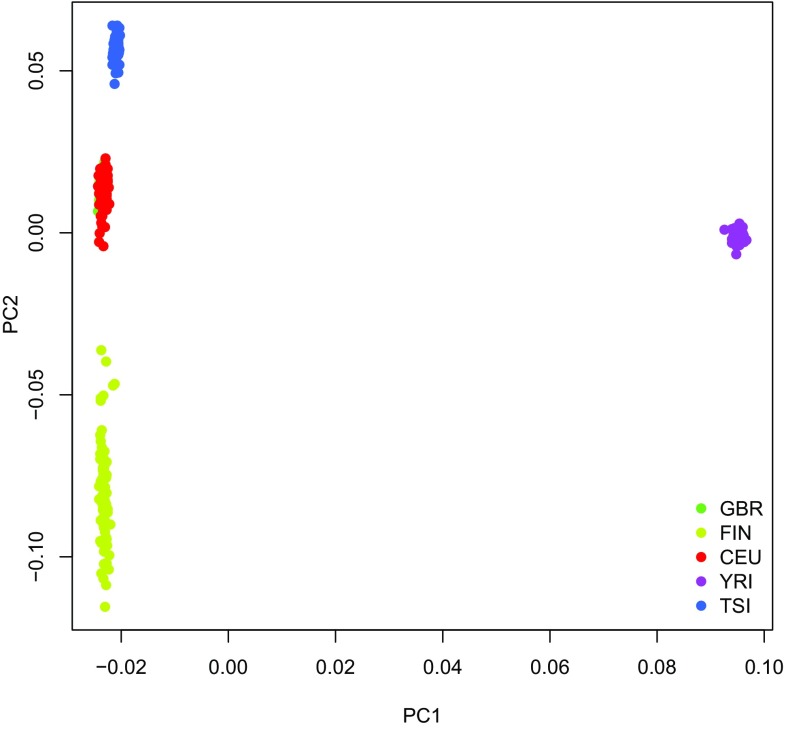
PCA plot of LD-pruned genome-wide SNP genotype data from the 1000 Genomes/GEUVADIS project. Dots were colored according to subgroup affiliation. *GBR* British, *FIN* Finns, *CEU* Utah residents with Northern and Western European ancestry, *YRI* Yoruba, *TSI* Tuscans

Principal component analysis (PCA) of the SNP genotypes from 1000 Genomes/ GEUVADIS revealed clear differences between the African (YRI) and the European ancestry subgroups (GBR, FIN, CEU, TSI; Fig. 2). The respective genotype clusters were widely separated along the 1st principal component (PC), which explained 10.5% of the variance in genotype. Differences between European ancestry subgroups only became apparent along the 2nd PC, which explained 0.6% of the variance.

When the metabolic network coherence (MC) values derived from the 1000 Genomes/ GEUVADIS transcriptome data were assessed for subgroup heterogeneity using a Kruskal–Wallis test, no statistically significant inter-population differences were found. However, when the combined European ancestry populations were compared to YRI, a difference verging on statistical significance became apparent (Wilcoxon test *p* = 0.067). To reduce the risk of confounding, the YRI data were, therefore, excluded from the subsequent GWAS. An additional analysis comprising both the YRI and the other 1000 Genomes/ GEUVADIS data was performed to check the robustness of the European ancestry-based results.

**Fig. 3:**
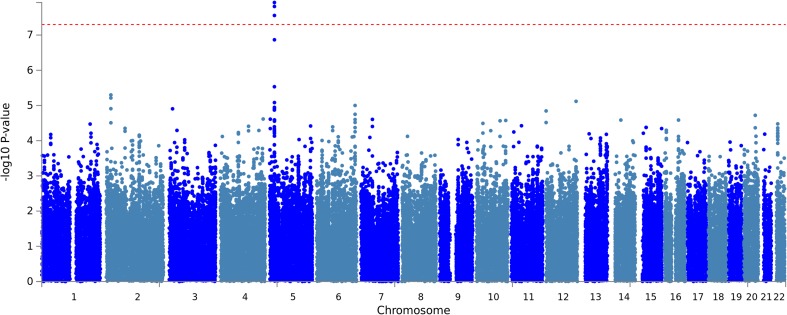
Manhattan plot of the primary GWAS of MC (1000 Genomes/ GEUVADIS, European ancestry subgroups only). The red line delineates genome-wide significance (i.e., *p* < 5 × 10^− 8^)

**Fig. 4:**
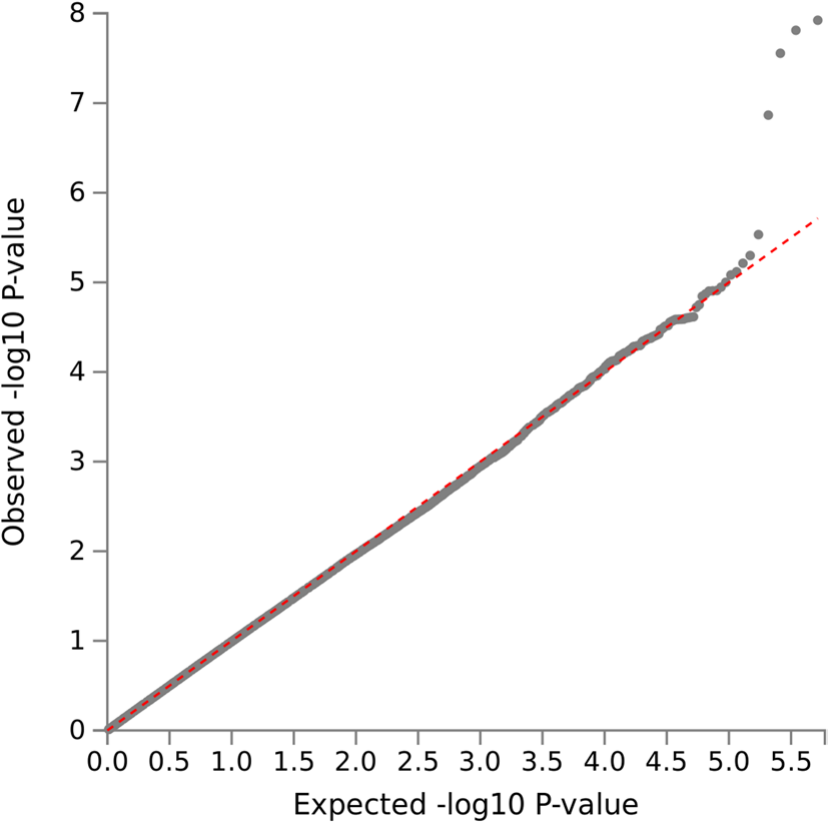
QQ plot of *p* values from the primary GWAS of MC (1000 Genomes/GEUVADIS, European ancestry subgroups only)

A GWAS of the individuals of European ancestry in the 1000 Genomes/ GEUVADIS study yielded a single genome-wide significant association with MC, located in the cadherin18 (*CDH18*) gene region on human chromosome 5 (top SNP: rs11744487, *p* = 1.2 × 10^− 8^). As can be inferred from the summary Manhattan plot (Fig. 3), no other genomic region showed an association with MC of similar significance. Moreover, the corresponding QQ-plot (Fig. 4) strongly suggests that the test statistics and, hence, the *p* values of the GWAS were not systematically inflated. The additional GWAS also including the YRI subgroup resulted in an MC association of the *CDH18* gene region that was still verging on genome-wide significance (top SNP rs11744487 *p* = 5.5 × 10^− 8^) and, as with the primary GWAS, no other signals above random noise were observed (Figs. S3, S4). Similarly, the top SNP showed a clear genotype dosage effect on MC (Fig. S5) as observed in the European-descent populations alone (see below).

The GWAS results were robust to alterations of the proportion of currency metabolites excluded and of the salient expression threshold used. Even although *p* values associated with the top SNPs were found to vary when the respective parameters were changed, the qualitative outcome remained the same, including the observation of a single association signal at the *CDH18* gene locus. Further details of the GWAS subgroup and sensitivity analyses can be found in Supplementary Figure S6 and Supplementary Table S2.

For three SNPs (rs11744487, rs1876591, rs925185) in the *CDH18* gene region, the association between genotype and MC was of genome-wide significance in the primary GWAS (*p* < 5 × 10^− 8^; Table 2). All three SNPs are located in introns of the *CDH18* gene. Markers rs925185 and rs1876591 were found to be in strong linkage disequilibrium (LD) with each other (*r*^2^ = 0.98), but in low LD with top SNP rs11744487 (*r*^2^ = 0.24 and *r*^2^ = 0.35, respectively). Therefore, they potentially represented a single albeit independent association with MC. Moreover, in additional analyses adjusted for the top SNP genotype, we detected 41 nominally significant MC associations of *CDH18* gene SNPs (*p* < 0.05), one of which withstood Bonferroni correction for the number of LD-effective SNPs in the region (*n* = 45, see below). The respective marker (rs4867798, *p* = 9.6 × 10^− 4^) is located at chr5:19677485, some 432 kb upstream of rs11744487 and 517 kb upstream of rs925185. The second and third smallest *p* values were obtained for the above-mentioned, genome-wide significant SNPs rs1876591 (*p* = 2.6 × 10^− 3^) and rs925185 (*p* = 2.7 × 10^− 3^), thereby supporting the conclusion that their MC association was largely independent of that of the top SNP.

**Table 2:** Statistically significant associations between MC and *CDH18* gene SNPs

Study	SNP	Location (bp)	Minor allele	Major allele	MAF	GWAS *p* value
1000 Genomes/GEUVADIS, European ancestry	rs11744487	chr5:20109561	A	T	0.49	1.2 × 10^− 8^
	rs1876591	chr5:20201563	C	G	0.49	1.5 × 10^− 8^
	rs925185	chr5:20194982	T	A	0.48	2.8 × 10^− 8^
BIOS	rs4866180	chr5:20328064	C	G	0.45	4.6 × 10^− 4^
	rs6884961	chr5:20325180	G	C	0.46	4.8 × 10^− 4^

*MAF* minor allele frequency

**Fig. 5:**
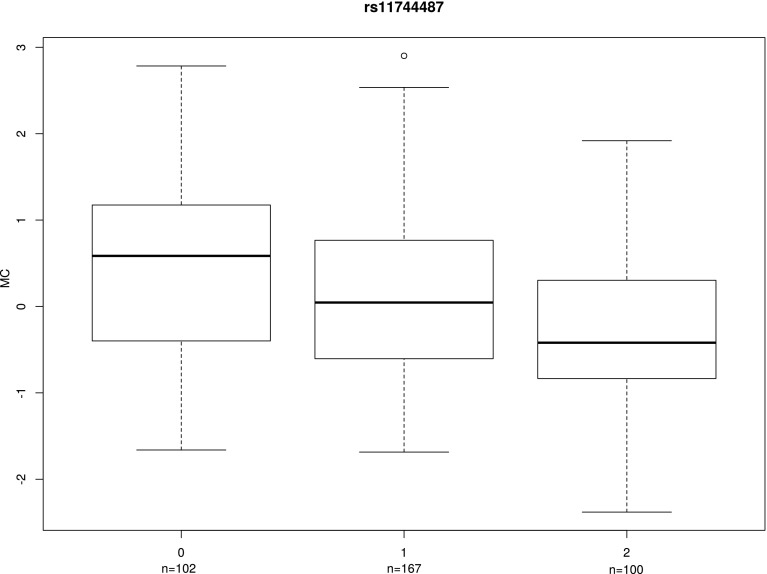
Dosage effect of rs11744487 genotype on MC in the 1000 Genomes/ GEUVADIS data (European ancestry only). Genotypes are encoded as 0: AA, 1: AT, 2: TT

A clear dosage effect on MC was noted for the genotype of GWAS top SNP rs11744487 (Fig. 5). While the highest median MC value was observed for homozygotes for minor allele A, and the lowest for TT homozygotes, AT heterozygotes showed intermediate MC.

**Fig. 6:**
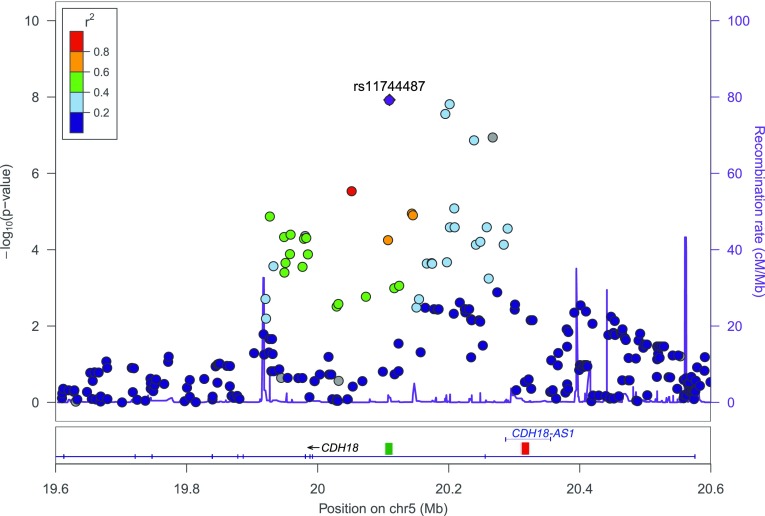
Locus zoom plot of GWAS results from the 1000 Genomes/GEUVADIS data (European ancestry only). Depicted is the 1 Mb region around the *CDH18* gene (chr5:19600000–chr5:20600000, based on hg19). The location of antisense RNA CDH18-AS1 is marked by a horizontal blue line whereas the lincRNA positions are indicated by squares (ENSG00000214132: green, ENSG00000248766: red). The top SNP (rs11744487) is highlighted in purple. Linkage disequilibrium is color-coded (red to blue) according to *r*^2^ with the top SNP

In the 1 Mb region surrounding the *CDH18* gene (chr5:19600000–chr5:20600000), 280 SNPs passed quality control in the 1000 Genomes/ GEUVADIS data (Fig. 6). The number of LD-effective SNPs in the region, as calculated from the same dataset, equaled 45. Based on the results of the primary GWAS, these 280 SNPs were chosen for replication of the putative MC association in the KORA, GENOA and BIOS studies. Notably, functional annotation of the target region with FUMA revealed the presence of two long non-coding RNAs (lncRNAs; ENSG00000214132, ENSG00000248766) and one antisense RNA (CDH18-AS1).

**Fig. 7:**
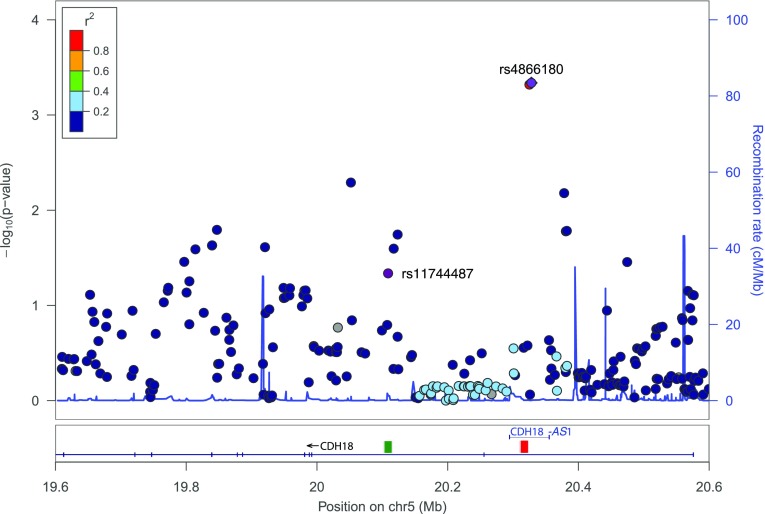
Locus-zoom plot of MC-SNP associations in the BIOS data. Depicted is the 1 Mb region around the *CDH18* gene (chr5:19600000–chr5:20600000, based on hg19). The location of antisense RNA CDH18-AS1 is marked by a horizontal blue line, whereas the lincRNA positions are indicated by squares (ENSG00000214132: green, ENSG00000248766: red). Both the top SNP from the replication analysis (rs4866180) and the top SNP from the primary GWAS (rs11744487) are highlighted in purple. Linkage disequilibrium is color-coded (red to blue) according to r^2^ with the top SNP from the replication analysis

Replication of the GWAS results failed in the KORA and GENOA studies in that neither rs11744787 nor any of the other SNPs in the *CHD18* gene region was significantly associated with MC (for further details, see Supplementary Figs. S7 and S8). In contrast, two SNPs (rs4866180, *p* = 4.6 × 10^− 4^; rs6884961; *p* = 4.8 × 10^− 4^) showed a statistically significant association in the BIOS study after Bonferroni correction for the number of LD-effective SNPs in the region (i.e., *p* < 0.001; Fig. 7; Table 2). These two SNPs were found to be in strong linkage disequilibrium with each other (*r*^2^ = 0.92), but not with rs11744487 (*r*^2^ = 0.01 for both rs4866180 and rs6884961). Notably, their association with MC was also verging on nominal significance in the 1000 Genomes/ GEUVADIS dataset (*p* = 0.061 for rs4866180, *p* = 0.072 for rs6884961). For a meta-analysis highlighting the robustness of the associations between MC and SNPs from the *CDH18* gene region, see Supplementary Table S3.

Closer inspection of the region showed that both SNPs are located at the center of antisense RNA CDH18-AS1, some 200 kb downstream of the GWAS top SNP (Fig. 7). Moreover, consultation of the Genotype-Tissue Expression (GTEx) database (The GTEx Consortium 2013) revealed that both SNPs act as cis-eQTLs of CDH18-AS1 in human testis, but not in brain (the other tissue in which the antisense RNA is expressed in human adults).

A trans-eQTL analysis of the 1348 Recon2 genes and the 280 SNPs from the *CDH18* gene region was carried out in the 1000 Genomes/ GEUVADIS dataset (European ancestry only) to elucidate whether the observed GWAS signal could be attributed to associations between the genotypes of particular SNPs and the expression levels of particular genes. However, while multiple SNP-specific use of a Kruskal–Wallis test revealed at least one nominally significant genotype-expression level association (*p* < 1.1 × 10^− 3^=0.05/45 upon correction for the number of LD-effective SNPs) for 96 genes (7.3% of Recon2 genes), none of these results withstood additional Bonferroni correction for the number of Recon2 genes tested (*n* = 1348; i.e., *p* < 8.2 × 10^− 7^). For a detailed summary of the results of the eQTL analysis, see Supplementary Table S4.

**Fig. 8:**
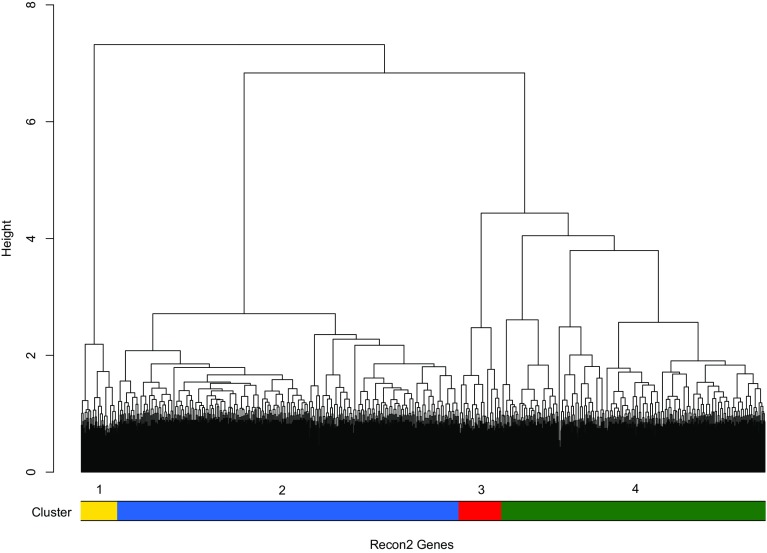
Cluster analysis of Recon2 gene expression levels in 1000 Genomes/GEUVADIS. The original RNA-seq data were dichotomized according to whether or not a particular expression value belonged to the gene-specific upper or lower 2% quantile in the 1000 Genomes/ GEUVADIS data. The four sub-clusters, color coded at the bottom of the dendrogram, were defined by reference to the gene-specific minimum *p* values obtained in a trans-eQTL analysis of the *CDH18* gene region (for details, see Methods)

The dichotomized expression values used for MC calculation (see Materials and Methods) were also subjected to hierarchical cluster analysis (Fig. 8) and the outcome was employed for a decomposition of the Recon2 genes into sub-clusters, based on the results of the eQTL analysis. To this end, the dendrogram height defining the number and identity of sub-clusters was gradually reduced. Each time when a sub-cluster was split into two new sub-clusters, a Kruskal–Wallis test was performed for the gene-specific minimum eQTL *p* values obtained in the *CDH18* gene region (see also Supplementary Table S4). The tests yielded a nominally significant result for four sub-clusters (*p* = 0.035; Fig. 8), but not for two (*p* = 0.105) or three sub-clusters (*p* = 0.171). Of the four sub-clusters, numbers 1 (72 genes) and 3 (84 genes) were characterized by a lower median *p* value (7.7 × 10^− 3^ and 7.5 × 10^− 3^, respectively) than numbers 2 (9.5 × 10^− 3^; 672 genes) and 4 (9.6 × 10^− 3^; 519 genes).

Analysis with DAVID 6.8 (Huang et al. 2009) yielded 23 biological terms that were found to be significantly enriched in sub-cluster 3 (*p* < 0.05 after Bonferroni correction; Table 3); no significantly enriched terms were reported for sub-cluster 1. The lowest enrichment *p* values were obtained for genes in sub-cluster 3 that are associated with KEGG terms related to neurodegenerative diseases (Huntington, Parkinson, Alzheimer), followed by several terms broadly related to energy metabolism and mitochondrial function.

**Table 3:** Biological theme enrichment analysis of 84 Recon2 genes with low minimum *p* values in a trans-eQTL analysis of the *CDH18* gene region (sub-cluster 3 in Fig. 8)

Category	Term	No. genes (%)^a^	Enrichment *p* value^b^
KEGG_PATHWAY	hsa05016: Huntington’s disease	34 (40)	1.2 × 10^− 19^
KEGG_PATHWAY	hsa05012: Parkinson’s disease	33 (39)	1.9 × 10^− 17^
KEGG_PATHWAY	hsa05010: Alzheimer’s disease	32 (38)	2.7 × 10^− 17^
GOTERM_CC_DIRECT	GO:0005743 ~ mitochondrial inner membrane	39 (46)	3.5 × 10^− 16^
UP_KEYWORDS	Acetylation	61 (73)	8.6 × 10^− 16^
UP_KEYWORDS	Mitochondrion inner membrane	34 (40)	1.3 × 10^− 15^
KEGG_PATHWAY	hsa00190: oxidative phosphorylation	34 (40)	1.5 × 10^− 15^
UP_KEYWORDS	Mitochondrion	50 (60)	1.4 ×10^− 13^
UP_KEYWORDS	Respiratory chain	21 (25)	1.9 × 10^− 11^
UP_KEYWORDS	Electron transport	22 (26)	1.7 × 10^− 10^
KEGG_PATHWAY	hsa04932: non-alcoholic fatty liver disease (NAFLD)	24 (29)	2.9 × 10^− 10^
GOTERM_MF_DIRECT	GO:0008137 ~ NADH dehydrogenase (ubiquinone) activity	16 (19)	4.2 × 10^− 8^
GOTERM_CC_DIRECT	GO:0005747 ~ mitochondrial respiratory chain complex I	16 (19)	2.5 × 10^− 8^
GOTERM_CC_DIRECT	GO:0005739 ~ mitochondrion	45 (54)	2.6 × 10^− 8^
GOTERM_BP_DIRECT	GO:0032981 ~ mitochondrial respiratory chain complex I assembly	16 (19)	1.6 × 10^− 7^
GOTERM_BP_DIRECT	GO:0006120 ~ mitochondrial electron transport. NADH to ubiquinone	16 (19)	2.3 × 10^− 7^
GOTERM_MF_DIRECT	GO:0044822 ~ poly(A) RNA binding	12 (14)	3.0 × 10^− 5^
UP_KEYWORDS	Transit peptide	29 (35)	3.9 × 10^− 5^
UP_SEQ_FEATURE	Transit peptide: mitochondrion	28 (33)	6.3 × 10^− 5^
GOTERM_CC_DIRECT	GO:0043209 ~ myelin sheath	14 (17)	5.4 × 10^− 5^
UP_KEYWORDS	Ubiquinone	11 (13)	2.3 × 10^− 4^
UP_KEYWORDS	Transport	36 (43)	1.3 × 10^− 3^
GOTERM_CC_DIRECT	GO:0070062 ~ extracellular exosome	41 (49)	7.6 × 10^− 3^

^a^Number of Recon2 genes associated with the term in an enrichment analysis with DAVID 6.8 (Huang et al. 2009)

^b^*p* values were Bonferroni corrected for the number of functional terms tested

## Discussion

We identified a single, genome-wide significant association between sequence variation at a particular gene locus and the metabolic coherence (MC) of human transcriptomes. The associated SNPs were located in the intronic region of the cadherin 18 (*CDH18*) gene on chromosome 5. This is a remarkable finding because GWAS of complex phenotypes usually lack such solitary and distinct signals but yield a number of significant associations instead, depending on the sample size as well as the genetic architecture and heritability of the trait under study. Although the 1000 Genomes/GEUVADIS sample was small compared to other recent GWAS, the peculiar distribution of *p* values observed in our study is not explicable by dearth of power alone. A lack of power would have resulted in a genome-wide gradient of signals, some of borderline statistical significance, rather than one protruding signal. Therefore, we have good reason to believe that the genome-wide significant association observed with MC points towards a genuine involvement of the *CDH18* gene locus in metabolic processes, at least as far as the expression of metabolism-relevant genes included in the Recon2 model is concerned.

Cadherin 18, previously termed cadherin 14, was first described by Shibata et al. (1997). It belongs to the large cadherin superfamily, a class of calcium-dependent trans-membrane proteins encoded by more than 100 genes (classical cadherins and related genes), many of which are organized in clusters. The cluster containing the *CDH18* gene is located on human chromosome 5 (5p14-15, 5q13-15 and 5q31-32) and deletions in this region have been linked to an increased risk of developing different diseases (Kajikawa et al. 2011; Zhang et al. 2013). Cadherins mediate cell–cell adhesion and play a vital role in tissue homeostasis and in morphogenesis (Leckband and Sivasankar 2012). For example, they regulate neural tube regionalization, neuronal migration, gray matter differentiation, neural circuit formation, spine morphology, synapse formation and synaptic plasticity (Redies et al. 2012). Furthermore, cadherins are also involved in intracellular signaling pathways.

Classical vertebrate cadherins are subdivided into type 1 and type 2, based on the presence of a histidine–alanine–valine motif in the first extracellular domain. Type 1 cadherins, which comprise some of the best characterized members of this class of proteins, are typically segregated by the embryonic germ layer or tissue type (Shapiro and Weis 2009). Type 2 cadherins like CDH18, in contrast, are less well characterized and exhibit more complex expression patterns, often associated with the developing neuronal system (Bekirov et al. 2002). Knockout of cadherin genes often leads to separation of cells or disrupts tissue architecture (Hirano and Takeichi 2012). On the other hand, overexpression of cadherin genes is frequently associated with the development of malignant tumors (Suyama et al. 2002). Noteworthy in the context of the present study, differential expression of cadherin genes has been observed in diseases with a metabolic component as well. For example, Burke et al. (2011) described upregulation of N-cadherin in fibroblasts from patients with Crohn disease, causing decreased wound-healing capacity and increased fibroblast migration. Similarly, a mutation in the E-cadherin gene was found to be associated with an increased risk of developing Crohn disease in the first place (Muise et al. 2009).

Cadherin 18 is expressed in various tissues, but appears to be confined mainly to the central nervous system (CNS; Bekirov et al. 2002). Consequently, *CDH18* gene expression itself was too low for reliable quantification in the datasets available for our study because the data were generated from either LCLs (1000 Genomes/ GEUVADIS, GENOA) or whole blood (KORA, BIOS). Since cadherin 18 is also not included in the Recon2 network, we suspect that the observed association between variation at the *CDH18* gene locus and MC is not due to any current activity of the cadherin 18 protein but involves other mechanisms, possibly during neuronal development, that govern metabolic processes in later life. In previous GWAS, variants of the *CDH18* gene were found to be associated with leprosy (Liu et al. 2015), age-related hearing impairment (Fransen et al. 2015), blood pressure-related traits in African-Americans (Liang et al. 2017) and with obesity in adult survivors of childhood cancer (Wilson et al. 2015). The last finding in particular suggests a role of the *CDH18* gene locus in body composition, with possible metabolic consequences during adulthood.

In a linkage study of adiponectin serving as a surrogate for metabolic syndrome, Comuzzie et al. (2001) identified genome-wide significant genetic linkage with the wider *CDH18* gene region at 5p14. Later, Zhang et al. (2013) explored the local effects further by fine mapping and discovered that several SNPs in the intronic region of *CDH18* were significantly associated with metabolic syndrome-related traits, including weight, BMI and waist circumference. The authors speculated that these associations were due to cell–cell adhesion processes, mediated by cadherin 18, with a direct impact upon the deposition of visceral abdominal fat reserves. Another possible explanation provided by Zhang et al. (2013) was a role of cadherin 18 in body development and body composition driven by the CNS during embryonic development.

A focused eQTL analysis in our study did not reveal any statistically significant associations between SNPs in the *CDH18* gene region and the expression of particular genes in Recon2, which implies that the genotypic association with MC that emerged in 1000 Genomes/ GEUVADIS and BIOS is not attributable to a small number of genes. This notwithstanding, when the Recon2 genes were clustered according to their degree of salient expression in the 1000 Genomes/GEUVADIS samples, those genes with the strongest association to variation at the *CDH18* locus were enriched for links to either neurodegenerative diseases or mitochondrial function. While the former clearly lends additional support to the hypothesis that the observed MC-*CDH18* association is driven by neurophysiological processes, the latter finding suggests that the downstream consequences of these processes may indeed include modifications of the energy metabolism in later life.

It must be emphasized here that the biological phenomenon underlying the GWAS signal detected in the 1000 Genomes/GEUVADIS and BIOS data, although not yet fully understood, would have gone unnoticed in a non-network-based eQTL analysis, particularly when carried out at genome-wide level and in moderately sized samples like those used in the our study. This outcome not only recalls the view of Carter et al. (2013) that many mutations exert their biological effects via network perturbation, but also highlights the potential benefit of adopting system-orientated approaches to molecular data analysis in general. They usually exploit the richness of data more comprehensively than classical analysis techniques and take the scientific value of the data themselves to a higher level by contextualizing them with external biological knowledge.

Prior to our validation analysis, we were concerned that replication of any potential GWAS signal from 1000 Genomes/GEUVADIS might fail in other studies if different cell types were used to measure gene expression. Whilst the 1000 Genomes/GEUVADIS and GENOA data were obtained from LCLs, KORA and BIOS used whole blood samples (Table 1). Indeed, the Epstein–Barr virus (EBV) transformation of LCLs is known to affect the expression of a number of genes, including those encoding cadherins (Breitfeld et al. 2016; Murakami et al. 2005), but because replication of the GWAS signal was successful in BIOS, not GENOA, EBV transformation can be ruled out as a cause of the observed discrepancies. Instead, it appears as if the technology used for expression analysis was more critical: In 1000 Genomes/GEUVADIS and BIOS, transcriptome data were generated by RNA-seq whereas microarrays were used in KORA and GENOA. Microarrays provide much lower genome coverage than RNA-seq, which in turn leads to a poorer representation of Recon2 genes in the datasets. Hence, the failed validation in KORA and GENOA may simply reflect a detection problem and the greater power of RNA-seq may be required to yield biologically meaningful results when applying network-based dimensionality reduction, such as MC, to whole transcriptome data.

Interestingly, top SNPs rs4866180 and rs6884961 from the successful replication analysis in BIOS are located in the center of an antisense RNA (CDH18-AS1). Moreover, both SNPs were found in GTEx to act as cis-eQTLs for CDH18-AS1 in humans. Although this effect was limited to testis, the only adult tissue apart from brain where CDH18-AS1 is expressed, our observation nevertheless suggests that *CDH18* gene variation may also play a regulatory role in other tissues during early human development as well. Hence, we are currently planning further laboratory experiments to determine if and how knockout or overexpression of cadherin 18 and CDH18-AS1 may impact upon metabolic processes in vitro.

## Electronic supplementary material

Below is the link to the electronic supplementary material.

Supplementary material 1 (PDF 1831 KB)
