# Supplementary material for: The metabolic network coherence of human transcriptomes is associated with genetic variation at the cadherin 18 locus
Source: Hum Genet. 2019 Mar 9;138(4):375–88. doi: 10.1007/s00439-019-01994-x (PMC6483969; doi:10.1007/s00439-019-01994-x)
Supplement: Supplementary file 1 — Supplementary material 1 (PDF 1831 KB) [file 439_2019_1994_MOESM1_ESM.pdf]

The metabolic network coherence of human transcriptomes is associated with genetic variation at the cadherin 18 locus (Schlicht, Nyzcka, et al.)

Supplementary Material

Part 1: Characteristics of datasets from four population-based studies

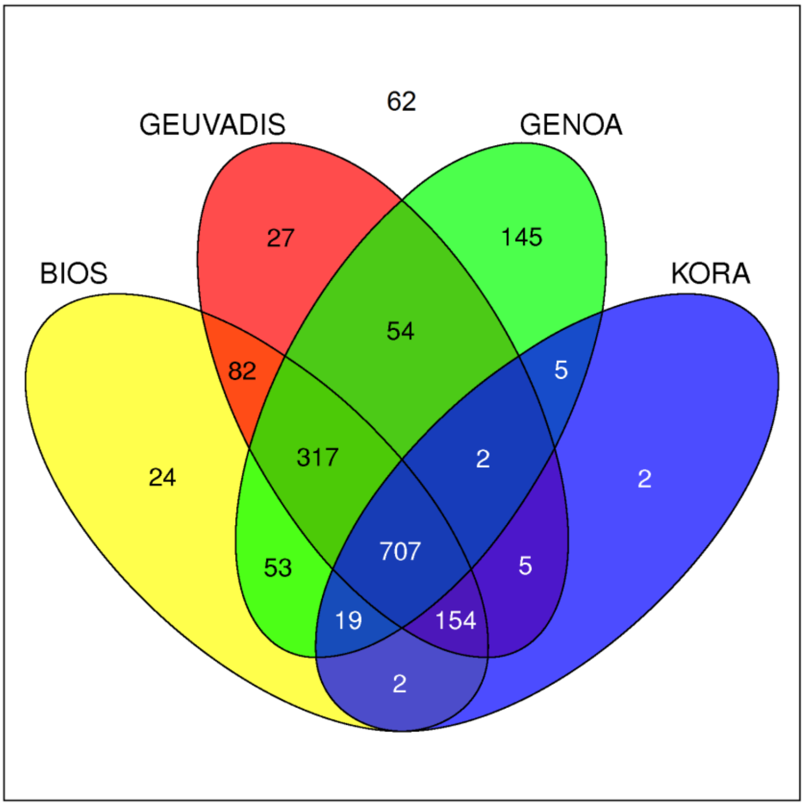

Fig. S1: Representation of Recon2 genes in transcriptome datasets. Note: A total of 1660 genes were included in the Recon2-derived gene-centric network used for MC calculation.

Table S1: Overlap of Recon2 genes present in different transcriptome datasets

	BIOS	GEUVADIS	GENOA	KORA
BIOS	-.-	0.63 (0.58-068)	0.12 (0.06-0.17)	0.63 (0.58-068)
GEUVADIS	0.89	-.-	0.08 (0.03-0.14)	0.36 (0.32-0.39)
GENOA	0.72	0.70	-.-	0.08 (0.03-0.12)
KORA	0.70	0.69	0.56	-.-

Upper right half: Cohen's kappa, with 95% confidence interval in round brackets; lower left half: pairwise concordance rate. All numbers were calculated with R package vcd.

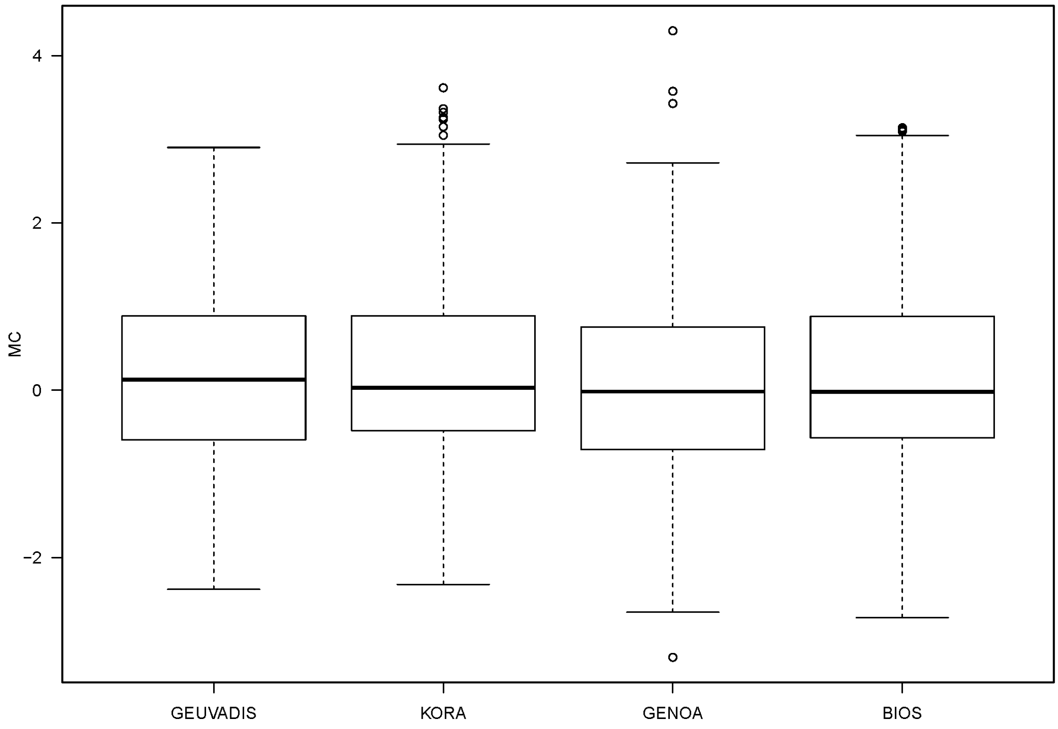

Fig. S2: Study-specific distribution of transcriptomic metabolic coherence (MC)

Part 2: GWAS of entire 1000Genomes/GEUVADIS dataset

An additional GWAS of MC was performed using the entire 1000Genomes/GEUVADIS dataset (YRI plus Europe ancestry subgroups). In agreement with the findings referred to in the main text, this analysis revealed highly suggestive associations only with SNPs from the *CDH18* gene region (Figs. S3 – S5).

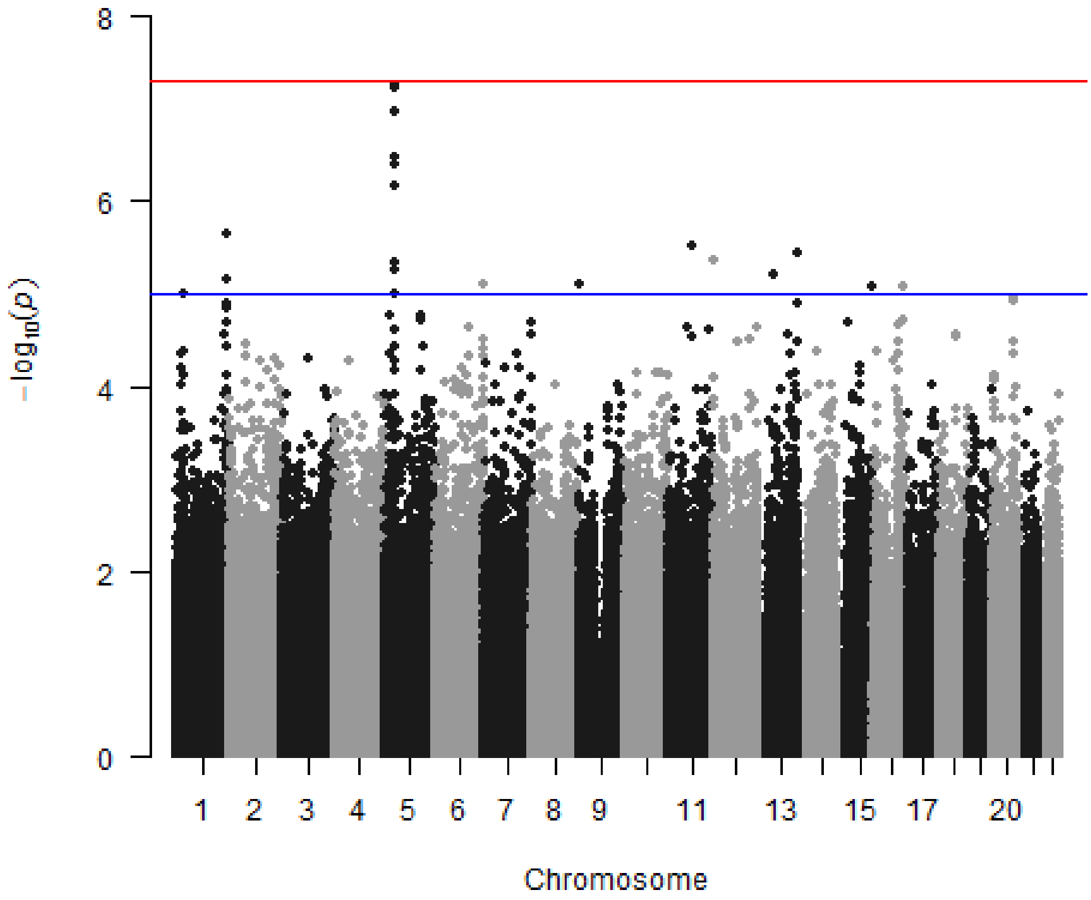

Fig. S3: Manhattan plot of GWAS in the 1000Genomes/GEUVADIS data, including YRI.

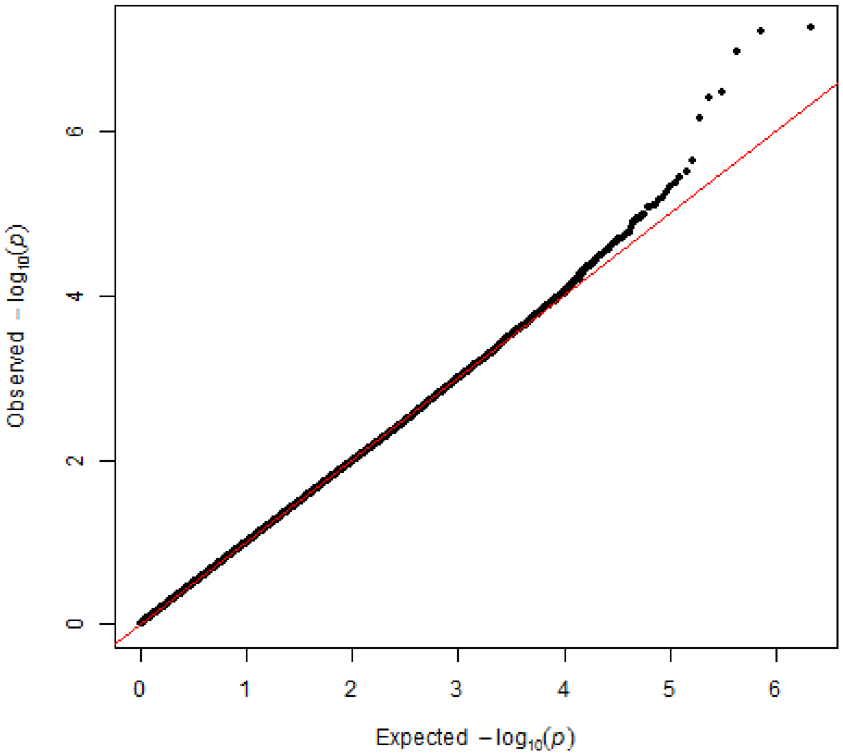

Fig. S4: QQ plot of GWAS in the 1000Genomes/GEUVADIS data, including YRI.

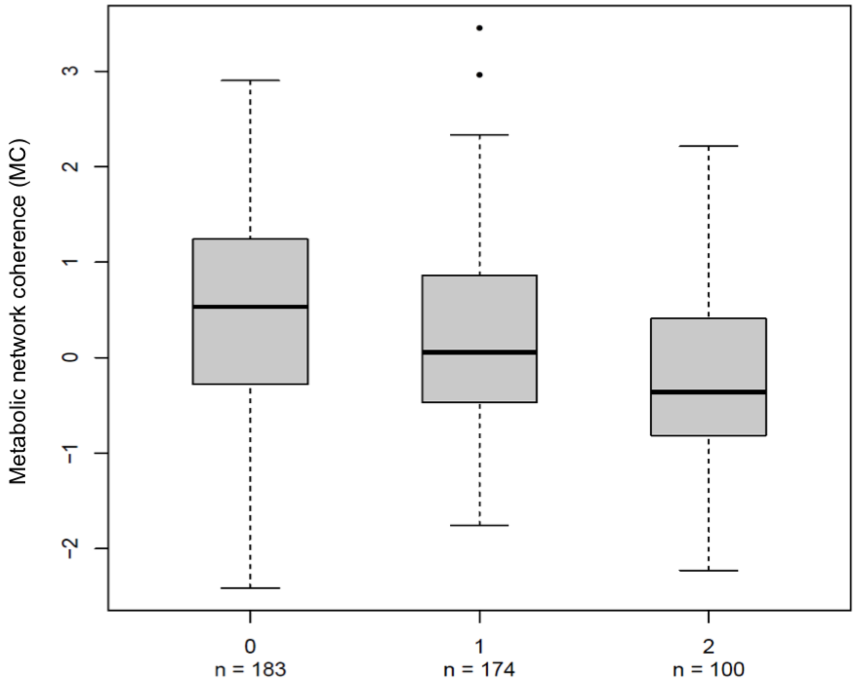

Fig. S5: Dosage effect of rs11744487 genotype on MC in the 1000Genomes/GEUVADIS data, including YRI. Genotypes are encoded as 0: AA, 1: AT, 2: TT.

Table S2: MC-SNP association of top GWAS SNPs in different 1000Genomes/ GEUVADIS population subgroups.

Population Subgroup	P value		
	rs11744487	rs1876591	rs925185
CEU	0.0485	0.0912	0.0912
FIN	0.0003	0.0055	0.0026
GBR	0.0011	0.0002	0.0002
TSI	0.0115	0.0052	0.0052
YRI	0.8519	0.4188	0.4465

Part 3: Sensitivity Analysis of GWAS in 1000 Genomes/ GEUVADIS data

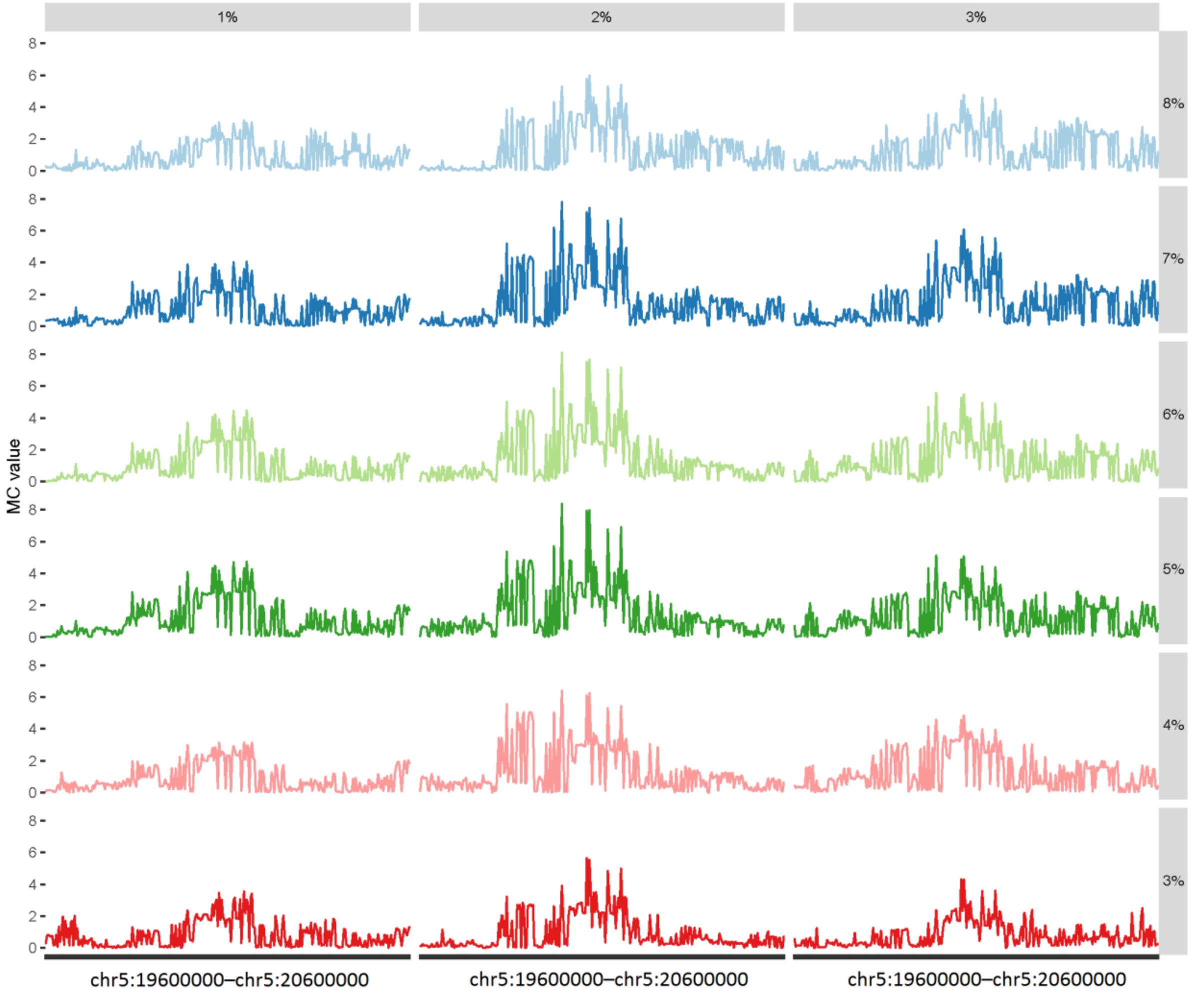

Fig. S6: Sensitivity analysis of MC GWAS, confined to the *CDH18* gene region, varying the proportion of currency metabolites excluded from the MC calculation (3%, bottom row, to 8%, top row) and the threshold for salient gene expression (1%, left column, to 3%, right column).

Part 4: MC-SNP associations in the *CDH18* region in KORA and GENOA

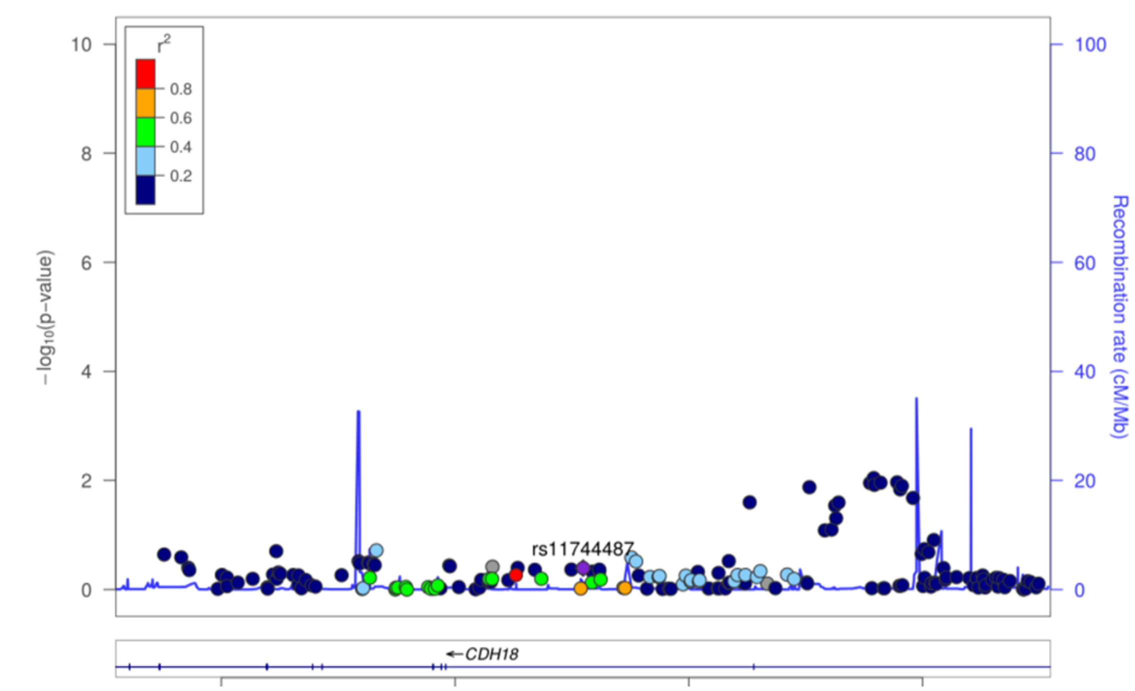

Fig. S7: Locus-zoom plot of MC-SNP associations in the KORA data. Depicted is the 1 Mb region around the *CDH18* gene (chr5:19600000–chr5:20600000, based upon hg19). The top SNP (r11744487) is highlighted in purple; linkage disequilibrium is color-coded (red to blue) according to r^2 with the top SNP.

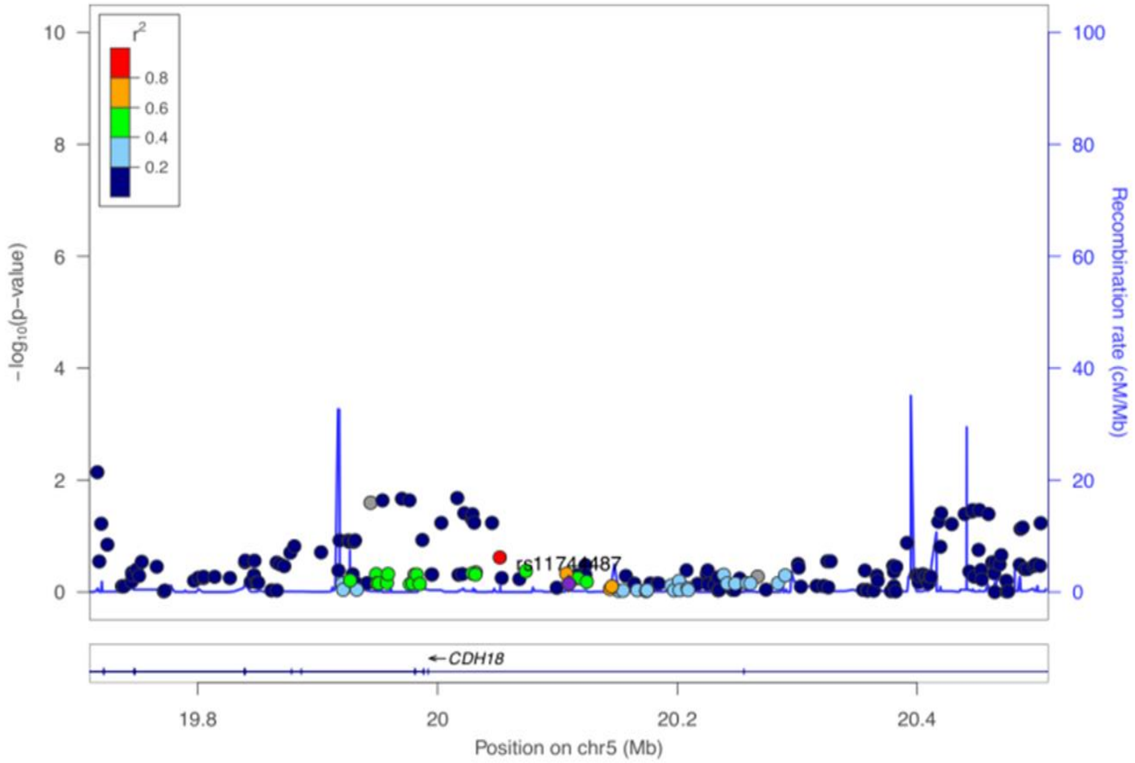

Fig. S8: Locus-zoom plot of MC-SNP associations in the GENOA data. For details, see legend to Fig. S7.

We carried out a p value-based meta-analysis of the *CDH18* gene region, using Fisher's chi-squared method as implemented in R package metap. As was to be expected, the meta-analysis p values were larger than the individual signals when 1000 Genome/ GEUVADIS and BIOS were combined (Table S3). What is more important, however, even when all four studies were included in the meta-analysis, the p values were still significant after Bonferroni correction for the number of LD-effective SNPs in the *CDH18* gene region (n=45).

Table S3: P value-based meta-analysis of MC association of selected *CDH18* SNPs

Studies	SNP			
	rs11744487	rs925185	rs4866180	rs6884961
All	2.3×10^{-7}	1.6×10^{-5}	1.8×10^{-5}	2.1×10^{-5}
GEUVADIS + BIOS	8.0×10^{-9}	4.0×10^{-7}	3.1×10^{-5}	3.2×10^{-5}

Part 5: BIOS Consortium (Biobank-based Integrative Omics Study) – Author information

Management Team Bastiaan T. Heijmans (chair)[1], Peter A.C. 't Hoen[2], Joyce van Meurs[3], Aaron Isaacs[4], Rick Jansen[5], Lude Franke[6].

Cohort collection Dorret I. Boomsma[7], René Pool[7], Jenny van Dongen[7], Jouke J. Hottenga[7] (Netherlands Twin Register); Marleen MJ van Greevenbroek[8], Coen D.A. Stehouwer[8], Carla J.H. van der Kallen[8], Casper G. Schalkwijk[8] (Cohort study on Diabetes and Atherosclerosis Maastricht); Cisca Wijmenga[6], Lude Franke[6], Sasha Zhernakova[6], Ettje F. Tigchelaar[6] (LifeLines Deep); P. Eline Slagboom[1], Marian Beekman[1], Joris Deelen[1], Diana van Heemst[9] (Leiden Longevity Study); Jan H.Veldink[10], Leonard H. van den Berg[10] (Prospective ALS Study Netherlands); Cornelia M. van Duijn[4],Bert A. Hofman[11], Aaron Isaacs[4], André G. Uitterlinden[3] (Rotterdam Study).

Data Generation Joyce van Meurs (Chair)[3], P. Mila Jhamai[3], Michael Verbiest[3], H. Eka D. Suchiman[1], Marijn Verkerk[3], Ruud van der Breggen[1], Jeroen van Rooij[3], Nico Lakenberg[1] .

Data management and computational infrastructure Hailiang Mei (Chair)[12], Maarten van Iterson[1], Michiel van Galen[2], Jan Bot[13], Dasha V. Zhernakova[6], Rick Jansen[5], Peter van 't Hof[12], Patrick Deelen[6], Irene Nooren[13], Peter A.C. 't Hoen[2], Bastiaan T. Heijmans[1], Matthijs Moed[1] .

Data Analysis Group Lude Franke (Co-Chair)[6], Martijn Vermaat[2], Dasha V. Zhernakova[6], René Luijk[1], Marc Jan Bonder[6], Maarten van Iterson[1], Patrick Deelen[6], Freerk van Dijk[14], Michiel van Galen[2], Wibowo Arindrarto[12], Szymon M. Kielbasa[15], Morris A. Swertz[14], Erik. W van Zwet[15], Rick Jansen[5], Peter-Bram 't Hoen (Co-Chair)[2], Bastiaan T. Heijmans (Co-Chair)[1].

1. Molecular Epidemiology Section, Department of Medical Statistics and Bioinformatics, Leiden University Medical Center, Leiden, The Netherlands
2. Department of Human Genetics, Leiden University Medical Center, Leiden, The Netherlands
3. Department of Internal Medicine, ErasmusMC, Rotterdam, The Netherlands
4. Department of Genetic Epidemiology, ErasmusMC, Rotterdam, The Netherlands
5. Department of Psychiatry, VU University Medical Center, Neuroscience Campus Amsterdam, Amsterdam, The Netherlands
6. Department of Genetics, University of Groningen, University Medical Centre Groningen, Groningen, The Netherlands
7. Department of Biological Psychology, VU University Amsterdam, Neuroscience Campus Amsterdam, Amsterdam, The Netherlands
8. Department of Internal Medicine and School for Cardiovascular Diseases (CARIM), Maastricht University Medical Center, Maastricht, The Netherlands
9. Department of Gerontology and Geriatrics, Leiden University Medical Center, Leiden, The Netherlands
10. Department of Neurology, Brain Center Rudolf Magnus, University Medical Center Utrecht, Utrecht, The Netherlands
11. Department of Epidemiology, ErasmusMC, Rotterdam, The Netherlands
12. Sequence Analysis Support Core, Leiden University Medical Center, Leiden, The Netherlands
13. SURFsara, Amsterdam, the Netherlands
14. Genomics Coordination Center, University Medical Center Groningen, University of Groningen,
Groningen, the Netherlands
15. Medical Statistics Section, Department of Medical Statistics and Bioinformatics, Leiden University, Medical Center, Leiden, The Netherlands

Part 6: eQTL Analysis

Table S4: Results of a trans-eQTL analysis of the 1000Genomes/GEUVADIS dataset (Europeans only). The analysis comprised the expression levels of 1348 Recon2 genes and the genotypes of 280 SNPs from the *CDH18* region of interest. Genes are ranked by the unadjusted p value obtained with a Kruskal-Wallis test. For genes where the expression level was found to be significantly associated with more than one SNP (p<0.05), the minimum p value is given. The sub-cluster assignment of Recon2 genes refers to Fig. 8 of the main text. No gene expression-SNP genotype association was statistically significant (p<0.05) after Bonferroni correction for the number of LD-efficient SNPs (n=45) and the number of Recon2 genes (n=1348).

**Gene**			**Minimum p value**	**SNP**
**Ensemble ID**	**HGNC symbol**	**Sub-cluster**		
ENSG00000197444	*OGDHL*	2	2.36E-05	rs10066903
ENSG00000104888	*SLC17A7*	2	2.80E-05	rs1498103
ENSG00000158669	*GPAT4*	2	5.35E-05	rs1909117
ENSG00000068976	*PYGM*	2	6.79E-05	rs1346543
ENSG00000117118	*SDHB*	4	7.38E-05	rs10066903
ENSG00000069849	*ATP1B3*	4	8.02E-05	rs10045245
ENSG00000129673	*AANAT*	2	1.13E-04	rs9292895
ENSG00000158006	*PAFAH2*	4	1.16E-04	rs34294319
ENSG00000185624	*P4HB*	4	1.33E-04	rs919344
ENSG00000110090	*CPT1A*	2	1.36E-04	rs7734813
ENSG00000154025	*SLC5A10*	4	1.56E-04	rs1508547
ENSG00000163931	*TKT*	1	1.67E-04	rs1909117
ENSG00000183077	*AFMID*	4	1.73E-04	rs1909117
ENSG00000136856	*SLC2A8*	2	1.79E-04	rs6888503
ENSG00000221988	*NA*	2	1.87E-04	rs7705221
ENSG00000131844	*MCCC2*	4	2.01E-04	rs11738280
ENSG00000084110	*HAL*	2	2.09E-04	rs13360869
ENSG00000181652	*ATG9B*	2	2.31E-04	rs61441877
ENSG00000152556	*PFKM*	4	2.37E-04	rs2202798
ENSG00000001084	*GCLC*	2	2.40E-04	rs164448
ENSG00000115919	*KYNU*	2	2.45E-04	rs1498103
ENSG00000008513	*ST3GAL1*	2	2.51E-04	rs12514159
ENSG00000023572	*GLRX2*	4	2.59E-04	rs10066903
ENSG00000204099	*NEU4*	2	2.64E-04	rs10066903
ENSG00000173175	*ADCY5*	2	2.65E-04	rs1347523
ENSG00000140905	*GCSH*	2	2.66E-04	rs919344
ENSG00000076555	*ACACB*	2	2.71E-04	rs12658322
ENSG00000116748	*AMPD1*	2	2.77E-04	rs10045715
ENSG00000162407	*PLPP3*	2	3.16E-04	rs7720726
ENSG00000100983	*GSS*	4	3.17E-04	rs3112489
ENSG00000161653	*NAGS*	1	3.21E-04	rs34294319
ENSG00000156110	*ADK*	3	3.23E-04	rs10066903
ENSG00000169299	*PGM2*	4	3.28E-04	rs62351320
ENSG00000148090	*AUH*	4	3.30E-04	rs1347523
ENSG00000153086	*ACMSD*	2	3.33E-04	rs10473357
ENSG00000126088	*UROD*	4	3.45E-04	rs10066903
ENSG00000149925	*ALDOA*	1	3.54E-04	rs4554223
ENSG00000160282	*FTCD*	2	3.55E-04	rs7716704
ENSG00000130304	*SLC27A1*	4	3.78E-04	rs1508547

ENSG00000184470	*TXNRD2*	4	3.79E-04	rs164448
ENSG00000023228	*NDUFS1*	4	3.80E-04	rs10066903
ENSG00000010932	*FMO1*	4	3.97E-04	rs72745083
ENSG00000083123	*BCKDHB*	3	3.97E-04	rs919344
ENSG00000172893	*DHCR7*	4	4.34E-04	rs4554223
ENSG00000100554	*ATP6V1D*	4	4.76E-04	rs62351320
ENSG00000166562	*SEC11C*	4	4.87E-04	rs4554223
ENSG00000111726	*CMAS*	3	5.02E-04	rs10045245
ENSG00000121900	*TMEM54*	2	5.20E-04	rs13360869
ENSG00000000419	*DPM1*	3	5.44E-04	rs10066903
ENSG00000187134	*AKR1C1*	2	5.53E-04	rs6884961
ENSG00000243989	*ACY1*	2	5.58E-04	rs13153197
ENSG00000137563	*GGH*	4	5.74E-04	rs10045245
ENSG00000158786	*PLA2G2F*	2	6.02E-04	rs10066903
ENSG00000103569	*AQP9*	2	6.08E-04	rs1508547
ENSG00000110435	*PDHX*	4	6.10E-04	rs58550676
ENSG00000183010	*PYCR1*	1	6.35E-04	rs73056793
ENSG00000104687	*GSR*	4	6.36E-04	rs35837974
ENSG00000117215	*PLA2G2D*	2	6.62E-04	rs7708358
ENSG00000142657	*PGD*	4	6.86E-04	rs1472892
ENSG00000138796	*HADH*	4	7.02E-04	rs7720726
ENSG00000154518	*ATP5MC3*	3	7.19E-04	rs73050988
ENSG00000143819	*EPHX1*	2	7.28E-04	rs10056397
ENSG00000145214	*DGKQ*	2	7.43E-04	rs9986203
ENSG00000140284	*SLC27A2*	2	7.45E-04	rs13175060
ENSG00000021826	*CPS1*	2	7.48E-04	rs7713439
ENSG00000132423	*COQ3*	3	7.48E-04	rs4288117
ENSG00000027847	*B4GALT7*	1	7.53E-04	rs16888249
ENSG00000118160	*SLC8A2*	4	7.65E-04	rs6884961
ENSG00000130035	*GALNT8*	2	7.65E-04	rs61441877
ENSG00000148154	*UGCG*	4	7.69E-04	rs10078676
ENSG00000076258	*FMO4*	2	7.74E-04	rs10073450
ENSG00000073417	*PDE8A*	4	7.75E-04	rs13170493
ENSG00000143772	*ITPKB*	2	7.84E-04	rs62351320
ENSG00000173540	*GMPPB*	4	7.99E-04	rs1911822
ENSG00000105220	*GPI*	4	7.99E-04	rs1823155
ENSG00000165195	*PIGA*	4	8.07E-04	rs73055416
ENSG00000205560	*CPT1B*	2	8.32E-04	rs4866190
ENSG00000074800	*ENO1*	3	8.53E-04	rs11745155
ENSG00000111640	*GAPDH*	4	8.57E-04	rs59277639
ENSG00000116459	*ATP5PB*	3	8.97E-04	rs4288117
ENSG00000079931	*MOXD1*	2	8.97E-04	rs7720726
ENSG00000079739	*PGM1*	3	9.11E-04	rs4866148
ENSG00000185100	*ADSSL1*	2	9.42E-04	rs4292450
ENSG00000115705	*TPO*	2	9.60E-04	rs13360869
ENSG00000113732	*ATP6V0E1*	4	9.75E-04	rs10045245
ENSG00000105281	*SLC1A5*	2	9.85E-04	rs10056397
ENSG00000132196	*HSD17B7*	4	9.86E-04	rs10070476
ENSG00000113790	*EHHADH*	4	9.89E-04	rs73050988
ENSG00000159593	*NAE1*	4	9.98E-04	rs4288117
ENSG00000141526	*SLC16A3*	1	1.01E-03	rs10941391
ENSG00000158089	*GALNT14*	2	1.04E-03	rs1703047
ENSG00000125430	*HS3ST3B1*	2	1.06E-03	rs10079950
ENSG00000179091	*CYC1*	4	1.07E-03	rs2115342
ENSG00000163738	*MTHFD2L*	2	1.09E-03	rs71610668
ENSG00000166411	*IDH3A*	4	1.09E-03	rs4288117
ENSG00000119523	*ALG2*	4	1.09E-03	rs2471154
ENSG00000151376	*ME3*	2	1.09E-03	rs2940461

ENSG00000078142	*PIK3C3*	4	1.10E-03	rs7736515
ENSG00000169418	*NPR1*	2	1.12E-03	rs9687957
ENSG00000138400	*MDH1B*	2	1.15E-03	rs59277639
ENSG00000124370	*MCEE*	2	1.16E-03	rs35738662
ENSG00000114054	*PCCB*	2	1.17E-03	rs13180247
ENSG00000169105	*CHST14*	4	1.17E-03	rs7720726
ENSG00000185875	*THNSL1*	2	1.18E-03	rs919344
ENSG00000129128	*SPCS3*	4	1.20E-03	rs4866148
ENSG00000112096	*SOD2*	4	1.20E-03	rs7736515
ENSG00000179163	*FUCA1*	2	1.20E-03	rs1472892
ENSG00000117394	*SLC2A1*	4	1.21E-03	rs62351320
ENSG00000168710	*AHCYL1*	2	1.22E-03	rs73056742
ENSG00000166016	*ABTB2*	2	1.23E-03	rs7708358
ENSG00000115339	*GALNT3*	2	1.24E-03	rs6863000
ENSG00000143315	*PIGM*	4	1.26E-03	rs11740264
ENSG00000066322	*ELOVL1*	4	1.26E-03	rs10066903
ENSG00000005022	*SLC25A5*	3	1.27E-03	rs73050988
ENSG00000100577	*GSTZ1*	2	1.27E-03	rs1909117
ENSG00000103876	*FAH*	2	1.28E-03	rs11952071
ENSG00000147383	*NSDHL*	3	1.28E-03	rs3112489
ENSG00000225697	*SLC26A6*	2	1.29E-03	rs10074201
ENSG00000100412	*ACO2*	2	1.29E-03	rs6864131
ENSG00000023330	*ALAS1*	3	1.30E-03	rs4288117
ENSG00000129219	*PLD2*	4	1.33E-03	rs1508547
ENSG00000140740	*UQCRC2*	3	1.35E-03	rs10066903
ENSG00000121310	*ECHDC2*	2	1.35E-03	rs6870586
ENSG00000197713	*RPE*	2	1.36E-03	rs6887118
ENSG00000151093	*OXSM*	4	1.36E-03	rs72745083
ENSG00000237289	*CKMT1B*	4	1.38E-03	rs1911822
ENSG00000082212	*ME2*	4	1.39E-03	rs10066903
ENSG00000131828	*PDHA1*	4	1.39E-03	rs16888249
ENSG00000152455	*SUV39H2*	4	1.42E-03	rs6451845
ENSG00000112972	*HMGCS1*	4	1.42E-03	rs4422533
ENSG00000169764	*UGP2*	4	1.43E-03	rs10070476
ENSG00000104812	*GYS1*	4	1.47E-03	rs1549653
ENSG00000101365	*IDH3B*	3	1.49E-03	rs10473357
ENSG00000172113	*NME6*	4	1.50E-03	rs10073450
ENSG00000111716	*LDHB*	3	1.51E-03	rs2202798
ENSG00000060642	*PIGV*	2	1.51E-03	rs189492
ENSG00000156515	*HK1*	4	1.53E-03	rs1823155
ENSG00000116171	*SCP2*	4	1.54E-03	rs73055416
ENSG00000137869	*CYP19A1*	2	1.55E-03	rs55724955
ENSG00000101945	*SUV39H1*	2	1.55E-03	rs7736515
ENSG00000166035	*LIPC*	2	1.56E-03	rs11956407
ENSG00000065911	*MTHFD2*	2	1.56E-03	rs73056793
ENSG00000124767	*GLO1*	3	1.57E-03	rs6872378
ENSG00000117594	*HSD11B1*	2	1.60E-03	rs10045245
ENSG00000151611	*MMAA*	2	1.62E-03	rs10045245
ENSG00000134333	*LDHA*	4	1.65E-03	rs1823155
ENSG00000054179	*ENTPD2*	2	1.65E-03	rs6884961
ENSG00000143554	*SLC27A3*	2	1.68E-03	rs73056763
ENSG00000162813	*BPNT1*	2	1.68E-03	rs13180247
ENSG00000067064	*IDI1*	4	1.70E-03	rs7734813
ENSG00000197121	*PGAP1*	2	1.71E-03	rs73055416
ENSG00000076685	*NT5C2*	2	1.71E-03	rs16888249
ENSG00000160211	*G6PD*	2	1.73E-03	rs7734813
ENSG00000140650	*PMM2*	2	1.73E-03	rs12153263
ENSG00000178035	*IMPDH2*	3	1.74E-03	rs73050988

ENSG00000204370	*SDHD*	2	1.75E-03	rs73050988
ENSG00000062282	*DGAT2*	2	1.76E-03	rs12517892
ENSG00000141446	*ESCO1*	4	1.76E-03	rs10069647
ENSG00000177542	*SLC25A22*	2	1.76E-03	rs73050988
ENSG00000140505	*CYP1A2*	2	1.76E-03	rs4400126
ENSG00000162174	*ASRGL1*	2	1.77E-03	rs6451573
ENSG00000176153	*GPX2*	2	1.77E-03	rs10066903
ENSG00000108846	*ABCC3*	2	1.78E-03	rs10941441
ENSG00000112394	*SLC16A10*	2	1.80E-03	rs1703042
ENSG00000177000	*MTHFR*	4	1.82E-03	rs13357704
ENSG00000119421	*NDUFA8*	3	1.83E-03	rs73050988
ENSG00000156973	*PDE6D*	4	1.83E-03	rs62351320
ENSG00000134824	*FADS2*	4	1.84E-03	rs4554223
ENSG00000206190	*ATP10A*	4	1.85E-03	rs1876603
ENSG00000100564	*PIGH*	2	1.85E-03	rs7736515
ENSG00000147853	*AK3*	4	1.86E-03	rs13360869
ENSG00000244038	*DDOST*	4	1.90E-03	rs10066903
ENSG00000039123	*MTREX*	4	1.92E-03	rs73055416
ENSG00000040933	*INPP4A*	4	1.92E-03	rs7713439
ENSG00000092009	*CMA1*	2	1.93E-03	rs10073450
ENSG00000104823	*ECH1*	1	1.93E-03	rs164448
ENSG00000147471	*PLPBP*	4	1.94E-03	rs72745083
ENSG00000074621	*SLC24A1*	4	1.94E-03	rs16888249
ENSG00000152952	*PLOD2*	2	1.98E-03	rs10045715
ENSG00000014641	*MDH1*	3	2.01E-03	rs73050988
ENSG00000099624	*ATP5F1D*	1	2.03E-03	rs16888249
ENSG00000099998	*GGT5*	2	2.04E-03	rs13357704
ENSG00000171097	*KYAT1*	2	2.10E-03	rs919344
ENSG00000181915	*ADO*	2	2.11E-03	rs2974591
ENSG00000100417	*PMM1*	2	2.11E-03	rs11738280
ENSG00000001630	*CYP51A1*	4	2.12E-03	rs7734813
ENSG00000068120	*COASY*	1	2.14E-03	rs6888503
ENSG00000143179	*UCK2*	4	2.16E-03	rs1498103
ENSG00000248144	*ADH1C*	2	2.20E-03	rs11738280
ENSG00000119227	*PIGZ*	4	2.23E-03	rs1823155
ENSG00000146477	*SLC22A3*	2	2.23E-03	rs6863000
ENSG00000174684	*B4GAT1*	2	2.25E-03	rs16888249
ENSG00000049239	*H6PD*	4	2.26E-03	rs10066903
ENSG00000126821	*SGPP1*	4	2.27E-03	rs992958
ENSG00000131373	*HACL1*	4	2.29E-03	rs73050988
ENSG00000133835	*HSD17B4*	4	2.29E-03	rs10066903
ENSG00000087008	*ACOX3*	4	2.30E-03	rs164448
ENSG00000109452	*INPP4B*	2	2.30E-03	rs35606542
ENSG00000164574	*GALNT10*	3	2.31E-03	rs1508547
ENSG00000097021	*ACOT7*	4	2.32E-03	rs7736515
ENSG00000112699	*GMDS*	2	2.32E-03	rs73050988
ENSG00000167283	*ATP5MG*	4	2.35E-03	rs62351320
ENSG00000172331	*BPGM*	4	2.35E-03	rs10045245
ENSG00000173599	*PC*	1	2.35E-03	rs1508547
ENSG00000083720	*OXCT1*	4	2.37E-03	rs1521026
ENSG00000107537	*PHYH*	2	2.40E-03	rs3112489
ENSG00000118520	*ARG1*	2	2.40E-03	rs7713439
ENSG00000170525	*PFKFB3*	2	2.43E-03	rs55749186
ENSG00000233276	*GPX1*	1	2.43E-03	rs16888249
ENSG00000157184	*CPT2*	4	2.45E-03	rs1472892
ENSG00000213614	*HEXA*	2	2.45E-03	rs17839229
ENSG00000113657	*DPYSL3*	2	2.53E-03	rs11741365
ENSG00000053371	*AKR7A2*	4	2.53E-03	rs7713439

ENSG00000179593	*ALOX15B*	2	2.54E-03	rs9687957
ENSG00000110911	*SLC11A2*	2	2.54E-03	rs13171040
ENSG00000157087	*ATP2B2*	2	2.54E-03	rs1508547
ENSG00000108439	*PNPO*	4	2.55E-03	rs1155040
ENSG00000142920	*AZIN2*	2	2.55E-03	rs4422533
ENSG00000183196	*CHST6*	2	2.55E-03	rs10076681
ENSG00000156136	*DCK*	4	2.55E-03	rs745105
ENSG00000163406	*SLC15A2*	2	2.57E-03	rs71610668
ENSG00000122729	*ACO1*	4	2.57E-03	rs6873205
ENSG00000075651	*PLD1*	2	2.58E-03	rs7720726
ENSG00000198931	*APRT*	1	2.60E-03	rs1703047
ENSG00000123360	*PDE1B*	4	2.62E-03	rs11952071
ENSG00000168575	*SLC20A2*	4	2.64E-03	rs1549653
ENSG00000100299	*ARSA*	2	2.64E-03	rs1911822
ENSG00000103202	*NME4*	2	2.66E-03	rs7713439
ENSG00000171608	*PIK3CD*	4	2.67E-03	rs4866148
ENSG00000152234	*ATP5F1A*	3	2.68E-03	rs1472892
ENSG00000176463	*SLCO3A1*	2	2.68E-03	rs10065518
ENSG00000109929	*SC5D*	4	2.68E-03	rs1549653
ENSG00000106733	*NMRK1*	2	2.68E-03	rs6888503
ENSG00000131748	*STARD3*	1	2.70E-03	rs6888503
ENSG00000095380	*NANS*	4	2.72E-03	rs73056793
ENSG00000154678	*PDE1C*	2	2.77E-03	rs7713439
ENSG00000107611	*CUBN*	2	2.79E-03	rs12153263
ENSG00000117450	*PRDX1*	3	2.79E-03	rs13357704
ENSG00000140374	*ETFA*	3	2.79E-03	rs73050988
ENSG00000093010	*COMT*	1	2.80E-03	rs16888249
ENSG00000167468	*GPX4*	1	2.81E-03	rs11745155
ENSG00000137700	*SLC37A4*	3	2.81E-03	rs1549653
ENSG00000105552	*BCAT2*	1	2.82E-03	rs7713439
ENSG00000151366	*NDUFC2*	1	2.82E-03	rs2136125
ENSG00000242110	*AMACR*	4	2.82E-03	rs1549653
ENSG00000146166	*LGSN*	2	2.82E-03	rs164448
ENSG00000083807	*SLC27A5*	4	2.83E-03	rs7708358
ENSG00000073849	*ST6GAL1*	4	2.83E-03	rs55749186
ENSG00000146733	*PSPH*	4	2.85E-03	rs7734813
ENSG00000139180	*NDUFA9*	2	2.86E-03	rs12514159
ENSG00000011083	*SLC6A7*	2	2.87E-03	rs75841406
ENSG00000051382	*PIK3CB*	2	2.88E-03	rs6872378
ENSG00000167114	*SLC27A4*	4	2.89E-03	rs6894851
ENSG00000010256	*UQCRC1*	3	2.93E-03	rs2892442
ENSG00000239900	*ADSL*	3	2.94E-03	rs1909117
ENSG00000110958	*PTGES3*	4	2.94E-03	rs10045245
ENSG00000122971	*ACADS*	1	2.94E-03	rs16888249
ENSG00000142892	*PIGK*	4	2.95E-03	rs189492
ENSG00000052802	*MSMO1*	4	2.97E-03	rs10066903
ENSG00000188687	*SLC4A5*	2	3.00E-03	rs6884683
ENSG00000100075	*SLC25A1*	1	3.01E-03	rs10941391
ENSG00000133116	*KL*	2	3.02E-03	rs11745155
ENSG00000105655	*ISYNA1*	2	3.03E-03	rs1352199
ENSG00000172831	*CES2*	2	3.04E-03	rs1549653
ENSG00000178234	*GALNT11*	2	3.05E-03	rs6884961
ENSG00000168282	*MGAT2*	4	3.06E-03	rs919344
ENSG00000126267	*COX6B1*	3	3.06E-03	rs13357704
ENSG00000100116	*GCAT*	4	3.09E-03	rs10065518
ENSG00000120437	*ACAT2*	4	3.10E-03	rs73050988
ENSG00000123505	*AMD1*	4	3.11E-03	rs10066903
ENSG00000169021	*UQCRFS1*	4	3.15E-03	rs73050988

ENSG00000119723	*COQ6*	2	3.15E-03	rs745105
ENSG00000124067	*SLC12A4*	2	3.16E-03	rs12658322
ENSG00000183696	*UPP1*	NA	3.18E-03	rs9687957
ENSG00000174876	*AMY1B*	4	3.19E-03	rs13360869
ENSG00000116141	*MARK1*	2	3.20E-03	rs1007844
ENSG00000103740	*ACSBG1*	4	3.20E-03	rs4288117
ENSG00000111846	*GCNT2*	2	3.21E-03	rs75841406
ENSG00000145391	*SETD7*	2	3.22E-03	rs7720726
ENSG00000169100	*SLC25A6*	4	3.28E-03	rs16888249
ENSG00000135740	*SLC9A5*	2	3.29E-03	rs62351320
ENSG00000130821	*SLC6A8*	4	3.29E-03	rs925185
ENSG00000131386	*GALNT15*	2	3.30E-03	rs58550676
ENSG00000196502	*SULT1A1*	2	3.30E-03	rs73055416
ENSG00000126264	*HCST*	4	3.31E-03	rs2434785
ENSG00000103257	*SLC7A5*	2	3.33E-03	rs10056397
ENSG00000103024	*NME3*	1	3.33E-03	rs16888249
ENSG00000105851	*PIK3CG*	2	3.36E-03	rs1508547
ENSG00000159423	*ALDH4A1*	4	3.36E-03	rs6872966
ENSG00000105607	*GCDH*	1	3.38E-03	rs7736515
ENSG00000063176	*SPHK2*	1	3.39E-03	rs16888249
ENSG00000204310	*NA*	4	3.43E-03	rs10068995
ENSG00000066926	*FECH*	4	3.44E-03	rs1508547
ENSG00000093134	*VNN3*	2	3.45E-03	rs6884961
ENSG00000150768	*DLAT*	4	3.49E-03	rs4288117
ENSG00000033627	*ATP6V0A1*	4	3.49E-03	rs9687957
ENSG00000169738	*DCXR*	4	3.52E-03	rs164448
ENSG00000160883	*HK3*	2	3.52E-03	rs12658322
ENSG00000088035	*ALG6*	4	3.54E-03	rs13357704
ENSG00000115758	*ODC1*	4	3.55E-03	rs1521026
ENSG00000165140	*FBP1*	2	3.55E-03	rs13360869
ENSG00000004864	*SLC25A13*	2	3.55E-03	rs10076681
ENSG00000106633	*GCK*	4	3.55E-03	rs17839229
ENSG00000167508	*MVD*	1	3.56E-03	rs16888249
ENSG00000186204	*CYP4F12*	2	3.58E-03	rs10068995
ENSG00000117643	*MAN1C1*	2	3.58E-03	rs1703047
ENSG00000102967	*DHODH*	2	3.59E-03	rs919344
ENSG00000122126	*OCRL*	4	3.59E-03	rs11952071
ENSG00000015532	*XYLT2*	2	3.60E-03	rs10073450
ENSG00000178802	*MPI*	4	3.60E-03	rs55724955
ENSG00000151224	*MAT1A*	2	3.61E-03	rs10941842
ENSG00000102743	*SLC25A15*	4	3.61E-03	rs7734813
ENSG00000159399	*HK2*	2	3.62E-03	rs62351320
ENSG00000166391	*MOGAT2*	2	3.63E-03	rs12517650
ENSG00000155189	*AGPAT5*	4	3.65E-03	rs6888503
ENSG00000237763	*AMY1A*	4	3.65E-03	rs13360869
ENSG00000173868	*PHOSPHO1*	2	3.68E-03	rs11952071
ENSG00000175003	*SLC22A1*	2	3.68E-03	rs13360869
ENSG00000090054	*SPTLC1*	2	3.69E-03	rs4554223
ENSG00000080493	*SLC4A4*	2	3.70E-03	rs1549653
ENSG00000086544	*ITPKC*	4	3.71E-03	rs919344
ENSG00000140465	*CYP1A1*	2	3.72E-03	rs1508547
ENSG00000143198	*MGST3*	4	3.72E-03	rs62355178
ENSG00000139505	*MTMR6*	4	3.73E-03	rs6887118
ENSG00000154305	*MIA3*	2	3.73E-03	rs62351320
ENSG00000084774	*CAD*	4	3.73E-03	rs73055416
ENSG00000135423	*GLS2*	2	3.76E-03	rs11741365
ENSG00000163864	*NMNAT3*	2	3.77E-03	rs13184938
ENSG00000165672	*PRDX3*	4	3.79E-03	rs10066903

ENSG00000119640	*ACYP1*	2	3.80E-03	rs12517892
ENSG00000182197	*EXT1*	2	3.81E-03	rs35738662
ENSG00000197563	*PIGN*	4	3.81E-03	rs1435983
ENSG00000185000	*DGAT1*	1	3.82E-03	rs10473307
ENSG00000086475	*SEPHS1*	4	3.82E-03	rs1521026
ENSG00000078295	*ADCY2*	2	3.83E-03	rs4866150
ENSG00000175711	*B3GNTL1*	4	3.83E-03	rs4400126
ENSG00000138449	*SLC40A1*	2	3.84E-03	rs2892442
ENSG00000140400	*MAN2C1*	4	3.84E-03	rs7716704
ENSG00000197894	*ADH5*	4	3.84E-03	rs73050988
ENSG00000229937	*PRPS1L1*	2	3.85E-03	rs2060533
ENSG00000123989	*CHPF*	2	3.87E-03	rs7721392
ENSG00000160200	*CBS*	2	3.88E-03	rs13170385
ENSG00000106397	*PLOD3*	2	3.90E-03	rs73050988
ENSG00000135454	*B4GALNT1*	2	3.91E-03	rs1876603
ENSG00000122643	*NT5C3A*	2	3.91E-03	rs2115342
ENSG00000108479	*GALK1*	1	3.94E-03	rs2078188
ENSG00000138801	*PAPSS1*	4	3.95E-03	rs67911776
ENSG00000140675	*SLC5A2*	2	3.95E-03	rs11738280
ENSG00000162368	*CMPK1*	4	3.97E-03	rs11748566
ENSG00000119125	*GDA*	2	3.98E-03	rs11740264
ENSG00000004779	*NDUFAB1*	4	3.99E-03	rs10045245
ENSG00000163344	*PMVK*	1	4.02E-03	rs3112489
ENSG00000088766	*CRLS1*	3	4.03E-03	rs71610668
ENSG00000113504	*SLC12A7*	2	4.05E-03	rs7713439
ENSG00000123836	*PFKFB2*	2	4.05E-03	rs10069647
ENSG00000204228	*NA*	4	4.05E-03	rs1823155
ENSG00000184752	*NDUFA12*	3	4.06E-03	rs62351320
ENSG00000142583	*SLC2A5*	2	4.09E-03	rs10066903
ENSG00000012232	*EXTL3*	4	4.10E-03	rs11740264
ENSG00000159899	*NPR2*	2	4.12E-03	rs13178644
ENSG00000100344	*PNPLA3*	2	4.12E-03	rs2136125
ENSG00000113231	*PDE8B*	2	4.14E-03	rs73055416
ENSG00000087111	*PIGS*	4	4.15E-03	rs10066903
ENSG00000146085	*MUT*	4	4.17E-03	rs6887118
ENSG00000165609	*NUDT5*	2	4.18E-03	rs62351320
ENSG00000131055	*COX4I2*	2	4.21E-03	rs11741365
ENSG00000006534	*ALDH3B1*	2	4.23E-03	rs10066903
ENSG00000110955	*ATP5F1B*	3	4.24E-03	rs4288117
ENSG00000100596	*SPTLC2*	2	4.26E-03	rs1549653
ENSG00000189043	*NDUFA4*	2	4.26E-03	rs73050988
ENSG00000198721	*ECI2*	2	4.26E-03	rs3112489
ENSG00000204084	*INPP5B*	4	4.26E-03	rs3112489
ENSG00000102172	*SMS*	4	4.27E-03	rs10045245
ENSG00000150787	*PTS*	2	4.27E-03	rs10066903
ENSG00000174233	*ADCY6*	2	4.29E-03	rs1508547
ENSG00000174886	*NDUFA11*	1	4.29E-03	rs16888249
ENSG00000105641	*SLC5A5*	2	4.29E-03	rs992958
ENSG00000152782	*PANK1*	4	4.31E-03	rs73056742
ENSG00000168906	*MAT2A*	4	4.32E-03	rs4292450
ENSG00000008130	*NADK*	1	4.34E-03	rs1703047
ENSG00000149541	*B3GAT3*	1	4.34E-03	rs1703047
ENSG00000133313	*CNDP2*	4	4.36E-03	rs1508547
ENSG00000205309	*NT5M*	1	4.36E-03	rs16888249
ENSG00000196511	*TPK1*	2	4.37E-03	rs10035105
ENSG00000128609	*NDUFA5*	3	4.37E-03	rs1521026
ENSG00000138185	*ENTPD1*	4	4.39E-03	rs10041937
ENSG00000103056	*SMPD3*	2	4.41E-03	rs7708358

ENSG00000158296	*SLC13A3*	2	4.42E-03	rs6887118
ENSG00000120254	*MTHFD1L*	2	4.45E-03	rs4288117
ENSG00000123643	*SLC36A1*	2	4.45E-03	rs7720726
ENSG00000023909	*GCLM*	4	4.48E-03	rs59821454
ENSG00000016391	*CHDH*	2	4.49E-03	rs4554223
ENSG00000173660	*UQCRH*	3	4.49E-03	rs7708358
ENSG00000002726	*AOC1*	2	4.50E-03	rs10941441
ENSG00000095321	*CRAT*	2	4.51E-03	rs10066903
ENSG00000163755	*HPS3*	2	4.52E-03	rs2974591
ENSG00000170502	*NUDT9*	4	4.55E-03	rs10066903
ENSG00000167434	*CA4*	2	4.55E-03	rs10805661
ENSG00000089472	*HEPH*	2	4.57E-03	rs11741365
ENSG00000171766	*GATM*	2	4.57E-03	rs6888503
ENSG00000164708	*PGAM2*	2	4.57E-03	rs10066903
ENSG00000116711	*PLA2G4A*	2	4.59E-03	rs11955587
ENSG00000066230	*SLC9A3*	2	4.60E-03	rs11745155
ENSG00000131979	*GCH1*	2	4.61E-03	rs62351320
ENSG00000241878	*PISD*	4	4.61E-03	rs1508547
ENSG00000105205	*CLC*	2	4.62E-03	rs9986203
ENSG00000002549	*LAP3*	2	4.63E-03	rs10045245
ENSG00000128951	*DUT*	3	4.63E-03	rs1521023
ENSG00000157680	*DGKI*	2	4.65E-03	rs1911822
ENSG00000091137	*SLC26A4*	4	4.66E-03	rs12109816
ENSG00000154930	*ACSS1*	2	4.66E-03	rs4422533
ENSG00000169692	*AGPAT2*	1	4.71E-03	rs3112489
ENSG00000197818	*SLC9A8*	4	4.71E-03	rs71610668
ENSG00000135318	*NT5E*	2	4.73E-03	rs1911845
ENSG00000143393	*PI4KB*	2	4.73E-03	rs1508547
ENSG00000197977	*ELOVL2*	2	4.75E-03	rs1508547
ENSG00000141469	*SLC14A1*	2	4.76E-03	rs6884961
ENSG00000012779	*ALOX5*	2	4.78E-03	rs1703045
ENSG00000177156	*TALDO1*	4	4.80E-03	rs7736515
ENSG00000163281	*GNPDA2*	4	4.81E-03	rs4554223
ENSG00000116096	*SPR*	2	4.83E-03	rs7736515
ENSG00000182858	*ALG12*	4	4.85E-03	rs1703052
ENSG00000166262	*FAM227B*	4	4.85E-03	rs4866148
ENSG00000138115	*CYP2C8*	4	4.86E-03	rs6887118
ENSG00000197355	*UAP1L1*	4	4.86E-03	rs4554223
ENSG00000176928	*GCNT4*	4	4.88E-03	rs6869352
ENSG00000162688	*AGL*	4	4.89E-03	rs11738280
ENSG00000067057	*PFKP*	4	4.90E-03	rs12659684
ENSG00000135094	*SDS*	2	4.96E-03	rs10941842
ENSG00000259431	*THTPA*	4	4.98E-03	rs1703052
ENSG00000108515	*ENO3*	2	4.99E-03	rs6870586
ENSG00000167325	*RRM1*	2	5.01E-03	rs10066903
ENSG00000198162	*MAN1A2*	4	5.05E-03	rs13190608
ENSG00000155368	*DBI*	3	5.07E-03	rs1521026
ENSG00000114491	*UMPS*	4	5.08E-03	rs10066903
ENSG00000136877	*FPGS*	4	5.10E-03	rs7713439
ENSG00000167130	*DOLPP1*	4	5.10E-03	rs73056793
ENSG00000070019	*GUCY2C*	2	5.14E-03	rs10079950
ENSG00000198130	*HIBCH*	3	5.15E-03	rs1521026
ENSG00000128918	*ALDH1A2*	2	5.20E-03	rs1508547
ENSG00000105675	*ATP4A*	2	5.20E-03	rs11740264
ENSG00000150540	*HNMT*	2	5.22E-03	rs7736515
ENSG00000131471	*AOC3*	2	5.26E-03	rs10073450
ENSG00000100938	*GMPR2*	2	5.26E-03	rs2115342
ENSG00000143224	*PPOX*	2	5.26E-03	rs7713439

ENSG00000205669	*ACOT6*	2	5.27E-03	rs11744487
ENSG00000035687	*ADSS*	4	5.28E-03	rs919344
ENSG00000153933	*DGKE*	4	5.29E-03	rs11952071
ENSG00000112893	*MAN2A1*	2	5.29E-03	rs58017518
ENSG00000077044	*DGKD*	2	5.30E-03	rs6870586
ENSG00000182890	*GLUD2*	4	5.31E-03	rs10066903
ENSG00000103253	*HAGHL*	2	5.33E-03	rs4866190
ENSG00000167969	*ECI1*	1	5.36E-03	rs16888249
ENSG00000008311	*AASS*	2	5.36E-03	rs13360869
ENSG00000105409	*ATP1A3*	2	5.37E-03	rs4866039
ENSG00000111962	*UST*	2	5.38E-03	rs3112489
ENSG00000166816	*LDHD*	2	5.38E-03	rs986251
ENSG00000164867	*NOS3*	4	5.45E-03	rs2060533
ENSG00000100330	*MTMR3*	4	5.45E-03	rs7734813
ENSG00000137857	*DUOX1*	2	5.46E-03	rs745105
ENSG00000152127	*MGAT5*	2	5.47E-03	rs1876603
ENSG00000106258	*CYP3A5*	4	5.47E-03	rs1549653
ENSG00000073756	*PTGS2*	2	5.49E-03	rs72745083
ENSG00000161905	*ALOX15*	2	5.51E-03	rs11739635
ENSG00000148218	*ALAD*	4	5.52E-03	rs13183491
ENSG00000003989	*SLC7A2*	2	5.53E-03	rs4866039
ENSG00000158864	*NDUFS2*	4	5.54E-03	rs4288117
ENSG00000124406	*ATP8A1*	4	5.56E-03	rs10073450
ENSG00000091483	*FH*	3	5.56E-03	rs4288117
ENSG00000139209	*SLC38A4*	2	5.58E-03	rs1396340
ENSG00000248098	*BCKDHA*	2	5.58E-03	rs4866039
ENSG00000177465	*ACOT4*	2	5.58E-03	rs12514159
ENSG00000116353	*MECR*	3	5.59E-03	rs7713439
ENSG00000196517	*SLC6A9*	2	5.60E-03	rs10056397
ENSG00000124253	*PCK1*	2	5.64E-03	rs4401574
ENSG00000147224	*PRPS1*	4	5.66E-03	rs4288117
ENSG00000166123	*GPT2*	2	5.70E-03	rs10078346
ENSG00000151665	*PIGF*	2	5.72E-03	rs11745155
ENSG00000254685	*FPGT*	2	5.73E-03	rs1909117
ENSG00000133731	*IMPA1*	4	5.81E-03	rs7721392
ENSG00000137825	*ITPKA*	2	5.83E-03	rs11745155
ENSG00000118298	*CA14*	2	5.85E-03	rs13360869
ENSG00000167600	*CYP2S1*	2	5.85E-03	rs62355178
ENSG00000153574	*RPIA*	3	5.86E-03	rs1521026
ENSG00000079459	*FDFT1*	4	5.88E-03	rs7734813
ENSG00000125505	*MBOAT7*	1	5.89E-03	rs10066903
ENSG00000170522	*ELOVL6*	2	5.90E-03	rs6877123
ENSG00000114268	*PFKFB4*	4	5.94E-03	rs7734813
ENSG00000144591	*GMPPA*	2	5.95E-03	rs59199016
ENSG00000160752	*FDPS*	2	5.95E-03	rs62355178
ENSG00000174502	*SLC26A9*	2	5.96E-03	rs13171040
ENSG00000256269	*HMBS*	4	6.01E-03	rs6887953
ENSG00000112759	*SLC29A1*	4	6.01E-03	rs72745083
ENSG00000186642	*PDE2A*	2	6.04E-03	rs35837974
ENSG00000151729	*SLC25A4*	4	6.05E-03	rs6887953
ENSG00000125246	*CLYBL*	4	6.06E-03	rs4422533
ENSG00000171314	*PGAM1*	2	6.06E-03	rs7734813
ENSG00000171310	*CHST11*	2	6.07E-03	rs1876603
ENSG00000145020	*AMT*	2	6.08E-03	rs2892442
ENSG00000132681	*ATP1A4*	2	6.09E-03	rs35837974
ENSG00000132837	*DMGDH*	2	6.13E-03	rs73056763
ENSG00000109107	*ALDOC*	4	6.14E-03	rs1823155
ENSG00000092964	*DPYSL2*	2	6.14E-03	rs2434785

ENSG00000087053	*MTMR2*	2	6.14E-03	rs1435983
ENSG00000159131	*GART*	4	6.15E-03	rs10066903
ENSG00000185133	*INPP5J*	2	6.18E-03	rs1703047
ENSG00000162433	*AK4*	4	6.19E-03	rs73050988
ENSG00000136143	*SUCLA2*	4	6.21E-03	rs10066903
ENSG00000091140	*DLD*	4	6.22E-03	rs10066903
ENSG00000165458	*INPPL1*	4	6.24E-03	rs164448
ENSG00000183023	*SLC8A1*	2	6.24E-03	rs13360869
ENSG00000132958	*TPTE2*	2	6.26E-03	rs11745155
ENSG00000161533	*ACOX1*	4	6.27E-03	rs10066903
ENSG00000173221	*GLRX*	4	6.27E-03	rs10045245
ENSG00000140459	*CYP11A1*	4	6.28E-03	rs6887118
ENSG00000175040	*CHST2*	2	6.30E-03	rs1521026
ENSG00000108602	*ALDH3A1*	2	6.32E-03	rs10473344
ENSG00000198754	*OXCT2*	2	6.36E-03	rs7734813
ENSG00000147684	*NDUFB9*	3	6.36E-03	rs4288117
ENSG00000167703	*SLC43A2*	2	6.37E-03	rs16888249
ENSG00000050438	*SLC4A8*	2	6.38E-03	rs6870271
ENSG00000136213	*CHST12*	2	6.38E-03	rs10066903
ENSG00000083444	*PLOD1*	4	6.40E-03	rs6872966
ENSG00000099194	*SCD*	2	6.50E-03	rs11744487
ENSG00000058668	*ATP2B4*	2	6.54E-03	rs7720726
ENSG00000139631	*CSAD*	2	6.54E-03	rs6870586
ENSG00000113141	*IK*	2	6.58E-03	rs2892442
ENSG00000166165	*CKB*	2	6.59E-03	rs1911845
ENSG00000166800	*LDHAL6A*	2	6.61E-03	rs1909117
ENSG00000134255	*CEPT1*	4	6.61E-03	rs7708358
ENSG00000088305	*DNMT3B*	4	6.62E-03	rs11959190
ENSG00000007350	*TKTL1*	2	6.63E-03	rs16885636
ENSG00000213760	*NA*	4	6.66E-03	rs1508547
ENSG00000166796	*LDHC*	4	6.66E-03	rs10066903
ENSG00000140297	*GCNT3*	2	6.66E-03	rs1508547
ENSG00000119673	*ACOT2*	4	6.67E-03	rs1911845
ENSG00000165629	*ATP5F1C*	3	6.70E-03	rs10066903
ENSG00000113552	*GNPDA1*	2	6.71E-03	rs7708358
ENSG00000137992	*DBT*	4	6.71E-03	rs10066903
ENSG00000187091	*PLCD1*	2	6.71E-03	rs1508547
ENSG00000125257	*ABCC4*	4	6.72E-03	rs10473357
ENSG00000196839	*ADA*	2	6.77E-03	rs16888249
ENSG00000240303	*ACAD11*	2	6.78E-03	rs7736515
ENSG00000175198	*PCCA*	4	6.79E-03	rs67911776
ENSG00000105953	*OGDH*	4	6.81E-03	rs10066903
ENSG00000138356	*AOX1*	2	6.85E-03	rs10473391
ENSG00000071967	*CYBRD1*	2	6.87E-03	rs1521026
ENSG00000161217	*PCYT1A*	4	6.92E-03	rs6884961
ENSG00000115020	*PIKFYVE*	4	6.96E-03	rs10073450
ENSG00000139629	*GALNT6*	2	6.97E-03	rs11955587
ENSG00000168291	*PDHB*	4	6.99E-03	rs13357704
ENSG00000135390	*ATP5MC2*	3	7.02E-03	rs6884683
ENSG00000103502	*CDIPT*	1	7.07E-03	rs1508547
ENSG00000134294	*SLC38A2*	4	7.07E-03	rs2078188
ENSG00000117054	*ACADM*	2	7.10E-03	rs2974591
ENSG00000107902	*LHPP*	2	7.12E-03	rs1703049
ENSG00000136881	*BAAT*	2	7.13E-03	rs3112489
ENSG00000117009	*KMO*	4	7.15E-03	rs10035105
ENSG00000126091	*ST3GAL3*	2	7.15E-03	rs7736515
ENSG00000102100	*SLC35A2*	4	7.19E-03	rs13170493
ENSG00000112294	*ALDH5A1*	4	7.20E-03	rs10066903

ENSG00000143882	*ATP6V1C2*	4	7.20E-03	rs10066903
ENSG00000184227	*ACOT1*	4	7.20E-03	rs16888249
ENSG00000197763	*TXNRD3*	2	7.24E-03	rs2940461
ENSG00000119514	*GALNT12*	2	7.25E-03	rs73055416
ENSG00000144908	*ALDH1L1*	2	7.28E-03	rs6869352
ENSG00000093217	*XYLB*	4	7.32E-03	rs7736515
ENSG00000145284	*SCD5*	4	7.36E-03	rs2254473
ENSG00000168653	*NDUFS5*	3	7.38E-03	rs6887953
ENSG00000185813	*PCYT2*	1	7.41E-03	rs7713439
ENSG00000124181	*PLCG1*	2	7.46E-03	rs1549653
ENSG00000257594	*GALNT4*	4	7.46E-03	rs13183491
ENSG00000167815	*PRDX2*	2	7.47E-03	rs4866047
ENSG00000062485	*CS*	2	7.48E-03	rs11959190
ENSG00000186115	*CYP4F2*	2	7.49E-03	rs1911845
ENSG00000131069	*ACSS2*	4	7.53E-03	rs11740264
ENSG00000068745	*IP6K2*	4	7.55E-03	rs12153263
ENSG00000103222	*ABCC1*	4	7.58E-03	rs13170493
ENSG00000134184	*GSTM1*	2	7.61E-03	rs13178644
ENSG00000149809	*TM7SF2*	1	7.65E-03	rs16888249
ENSG00000100294	*MCAT*	1	7.66E-03	rs7734813
ENSG00000167315	*ACAA2*	3	7.67E-03	rs73050988
ENSG00000118402	*ELOVL4*	2	7.68E-03	rs11740264
ENSG00000154723	*ATP5PF*	3	7.69E-03	rs1521026
ENSG00000152463	*OLAH*	2	7.69E-03	rs13180247
ENSG00000151726	*ACSL1*	4	7.69E-03	rs10066903
ENSG00000182902	*SLC25A18*	2	7.70E-03	rs16885636
ENSG00000122863	*CHST3*	2	7.73E-03	rs11740264
ENSG00000173638	*SLC19A1*	1	7.78E-03	rs7721392
ENSG00000166228	*PCBD1*	4	7.79E-03	rs7720726
ENSG00000115425	*PECR*	2	7.80E-03	rs28648852
ENSG00000136908	*DPM2*	4	7.82E-03	rs11738280
ENSG00000171320	*ESCO2*	4	7.85E-03	rs919344
ENSG00000103356	*EARS2*	4	7.86E-03	rs12514159
ENSG00000137124	*ALDH1B1*	4	7.87E-03	rs10066903
ENSG00000104267	*CA2*	2	7.87E-03	rs9292940
ENSG00000171124	*FUT3*	4	7.91E-03	rs1521026
ENSG00000103489	*XYLT1*	2	7.92E-03	rs11748566
ENSG00000111713	*GYS2*	2	7.92E-03	rs17222830
ENSG00000116649	*SRM*	4	7.96E-03	rs7734813
ENSG00000149527	*PLCH2*	2	7.97E-03	rs1909117
ENSG00000175564	*UCP3*	2	7.98E-03	rs12109816
ENSG00000092621	*PHGDH*	2	8.09E-03	rs11739635
ENSG00000110719	*TCIRG1*	1	8.11E-03	rs17839229
ENSG00000102575	*ACP5*	2	8.13E-03	rs10073450
ENSG00000116133	*DHCR24*	4	8.14E-03	rs13357704
ENSG00000171759	*PAH*	2	8.15E-03	rs13360869
ENSG00000135845	*PIGC*	2	8.18E-03	rs3112489
ENSG00000124155	*PIGT*	4	8.19E-03	rs12652510
ENSG00000103485	*QPRT*	4	8.19E-03	rs164448
ENSG00000108784	*NAGLU*	1	8.22E-03	rs1508547
ENSG00000139514	*SLC7A1*	4	8.23E-03	rs4401574
ENSG00000198246	*SLC29A3*	2	8.27E-03	rs13170493
ENSG00000174951	*FUT1*	2	8.28E-03	rs7720726
ENSG00000113161	*HMGCR*	2	8.29E-03	rs7736515
ENSG00000121053	*EPX*	2	8.29E-03	rs10035105
ENSG00000100288	*CHKB*	2	8.31E-03	rs10065518
ENSG00000241468	*ATP5MF*	4	8.31E-03	rs72745083
ENSG00000197858	*GPAA1*	1	8.32E-03	rs16888249

ENSG00000161267	*BDH1*	2	8.33E-03	rs7713439
ENSG00000038002	*AGA*	4	8.38E-03	rs10066903
ENSG00000164405	*UQCRQ*	3	8.39E-03	rs4288117
ENSG00000156958	*GALK2*	2	8.42E-03	rs10066903
ENSG00000197142	*ACSL5*	4	8.48E-03	rs1521026
ENSG00000106348	*IMPDH1*	1	8.50E-03	rs10070476
ENSG00000139988	*RDH12*	2	8.52E-03	rs12659684
ENSG00000128059	*PPAT*	4	8.53E-03	rs10066903
ENSG00000140279	*DUOX2*	4	8.55E-03	rs9986203
ENSG00000138363	*ATIC*	4	8.56E-03	rs4288117
ENSG00000070961	*ATP2B1*	2	8.57E-03	rs16888249
ENSG00000132874	*SLC14A2*	4	8.57E-03	rs10941842
ENSG00000112695	*COX7A2*	4	8.57E-03	rs7708358
ENSG00000128309	*MPST*	2	8.59E-03	rs1911845
ENSG00000156471	*PTDSS1*	4	8.59E-03	rs10066903
ENSG00000143753	*DEGS1*	2	8.60E-03	rs10045245
ENSG00000119013	*NDUFB3*	3	8.60E-03	rs73050988
ENSG00000176095	*IP6K1*	4	8.61E-03	rs2202798
ENSG00000261052	*SULT1A3*	4	8.63E-03	rs13188919
ENSG00000108528	*SLC25A11*	4	8.63E-03	rs7736515
ENSG00000081760	*AACS*	2	8.64E-03	rs10473357
ENSG00000159267	*HLCS*	2	8.66E-03	rs57598264
ENSG00000146648	*EGFR*	2	8.69E-03	rs13180247
ENSG00000180011	*ZADH2*	4	8.79E-03	rs1911845
ENSG00000123983	*ACSL3*	4	8.79E-03	rs4288117
ENSG00000072756	*TRNT1*	4	8.81E-03	rs4288117
ENSG00000075415	*SLC25A3*	2	8.82E-03	rs57598264
ENSG00000072682	*P4HA2*	2	8.82E-03	rs1155040
ENSG00000171848	*RRM2*	4	8.82E-03	rs10079950
ENSG00000100253	*MIOX*	2	8.83E-03	rs7705221
ENSG00000104549	*SQLE*	2	8.85E-03	rs4866039
ENSG00000164889	*SLC4A2*	2	8.87E-03	rs7708358
ENSG00000100197	*CYP2D6*	2	8.88E-03	rs6883590
ENSG00000146701	*MDH2*	3	8.90E-03	rs16888249
ENSG00000168003	*SLC3A2*	2	8.92E-03	rs9687957
ENSG00000185527	*PDE6G*	2	8.92E-03	rs4422533
ENSG00000176387	*HSD11B2*	2	8.93E-03	rs7734813
ENSG00000121691	*CAT*	2	8.93E-03	rs6869352
ENSG00000185883	*ATP6V0C*	2	8.96E-03	rs55749186
ENSG00000167165	*UGT1A6*	2	8.98E-03	rs4866038
ENSG00000111181	*SLC6A12*	2	9.04E-03	rs1911822
ENSG00000159348	*CYB5R1*	4	9.05E-03	rs10066903
ENSG00000135929	*CYP27A1*	2	9.05E-03	rs7720726
ENSG00000176890	*TYMS*	4	9.09E-03	rs1521023
ENSG00000121281	*ADCY7*	2	9.10E-03	rs1703047
ENSG00000171174	*RBKS*	4	9.11E-03	rs2089556
ENSG00000113163	*COL4A3BP*	4	9.12E-03	rs1508547
ENSG00000160194	*NDUFV3*	4	9.14E-03	rs6869352
ENSG00000187733	*AMY1C*	4	9.15E-03	rs10069647
ENSG00000205060	*SLC35B4*	2	9.15E-03	rs12659684
ENSG00000163902	*RPN1*	4	9.16E-03	rs10045245
ENSG00000110080	*ST3GAL4*	2	9.17E-03	rs2078188
ENSG00000171503	*ETFDH*	4	9.24E-03	rs10056397
ENSG00000054148	*PHPT1*	1	9.24E-03	rs16888249
ENSG00000115286	*NDUFS7*	1	9.24E-03	rs16888249
ENSG00000047230	*CTPS2*	2	9.26E-03	rs1911822
ENSG00000180953	*ST20*	4	9.27E-03	rs55749186
ENSG00000012660	*ELOVL5*	2	9.28E-03	rs2471114

ENSG00000197943	*PLCG2*	4	9.31E-03	rs16888249
ENSG00000102032	*RENBP*	2	9.31E-03	rs16888249
ENSG00000131389	*SLC6A6*	2	9.33E-03	rs1549653
ENSG00000075239	*ACAT1*	3	9.36E-03	rs1435971
ENSG00000084754	*HADHA*	4	9.38E-03	rs6884683
ENSG00000100243	*CYB5R3*	2	9.43E-03	rs10069647
ENSG00000013375	*PGM3*	2	9.46E-03	rs7734813
ENSG00000152270	*PDE3B*	2	9.47E-03	rs1521026
ENSG00000140835	*CHST4*	2	9.49E-03	rs4404681
ENSG00000187210	*GCNT1*	4	9.49E-03	rs1508547
ENSG00000033100	*CHPF2*	2	9.49E-03	rs10473307
ENSG00000171793	*CTPS1*	4	9.54E-03	rs10065518
ENSG00000147606	*SLC26A7*	2	9.56E-03	rs2471148
ENSG00000155850	*SLC26A2*	2	9.62E-03	rs1909117
ENSG00000131238	*PPT1*	4	9.64E-03	rs10066903
ENSG00000152904	*GGPS1*	4	9.65E-03	rs13360869
ENSG00000106688	*SLC1A1*	2	9.67E-03	rs10045245
ENSG00000047249	*ATP6V1H*	4	9.70E-03	rs2471114
ENSG00000134575	*ACP2*	2	9.72E-03	rs11745155
ENSG00000135702	*CHST5*	2	9.73E-03	rs1521026
ENSG00000113924	*HGD*	2	9.73E-03	rs13170385
ENSG00000148288	*GBGT1*	2	9.73E-03	rs10473344
ENSG00000250565	*ATP6V1E2*	4	9.74E-03	rs55724955
ENSG00000174227	*PIGG*	2	9.75E-03	rs13360869
ENSG00000111275	*ALDH2*	2	9.76E-03	rs11738280
ENSG00000101444	*AHCY*	4	9.77E-03	rs919344
ENSG00000163655	*GMPS*	3	9.77E-03	rs1508582
ENSG00000074416	*MGLL*	2	9.78E-03	rs7713439
ENSG00000100031	*GGT1*	2	9.81E-03	rs10473357
ENSG00000172817	*CYP7B1*	2	9.85E-03	rs1549653
ENSG00000090013	*BLVRB*	2	9.90E-03	rs1508547
ENSG00000160408	*ST6GALNAC6*	2	9.91E-03	rs1549653
ENSG00000173614	*NMNAT1*	4	9.92E-03	rs11740264
ENSG00000102393	*GLA*	4	9.92E-03	rs10472421
ENSG00000151790	*TDO2*	2	9.94E-03	rs2940461
ENSG00000105679	*GAPDHS*	2	9.95E-03	rs10056397
ENSG00000125356	*NDUFA1*	3	9.97E-03	rs7708358
ENSG00000132746	*ALDH3B2*	2	9.97E-03	rs11952071
ENSG00000241343	*RPL36A*	3	9.99E-03	rs4554223
ENSG00000213930	*GALT*	2	1.00E-02	rs73055416
ENSG00000131174	*COX7B*	4	1.00E-02	rs1521026
ENSG00000060982	*BCAT1*	4	1.00E-02	rs35738662
ENSG00000130957	*FBP2*	4	1.00E-02	rs1911845
ENSG00000142168	*SOD1*	3	1.00E-02	rs1703047
ENSG00000185973	*TMLHE*	4	1.00E-02	rs10076681
ENSG00000186009	*ATP4B*	2	1.00E-02	rs7708358
ENSG00000048392	*RRM2B*	4	1.01E-02	rs4392624
ENSG00000143398	*PIP5K1A*	4	1.01E-02	rs7721392
ENSG00000064651	*SLC12A2*	4	1.01E-02	rs925185
ENSG00000169814	*BTD*	2	1.02E-02	rs1703047
ENSG00000133460	*SLC2A11*	2	1.02E-02	rs16885636
ENSG00000213398	*LCAT*	2	1.02E-02	rs6884961
ENSG00000123684	*LPGAT1*	4	1.02E-02	rs7708358
ENSG00000141429	*GALNT1*	4	1.03E-02	rs4400126
ENSG00000130816	*DNMT1*	4	1.03E-02	rs4866177
ENSG00000064225	*ST3GAL6*	2	1.03E-02	rs10079950
ENSG00000116337	*AMPD2*	2	1.03E-02	rs13157255
ENSG00000130377	*ACSBG2*	2	1.03E-02	rs11952071

ENSG00000106605	*BLVRA*	3	1.04E-02	rs13188919
ENSG00000007171	*NOS2*	2	1.04E-02	rs71610668
ENSG00000197217	*ENTPD4*	4	1.05E-02	rs16888249
ENSG00000115896	*PLCL1*	2	1.05E-02	rs4292450
ENSG00000132744	*ACY3*	2	1.06E-02	rs7705221
ENSG00000148672	*GLUD1*	4	1.06E-02	rs55822426
ENSG00000119927	*GPAM*	4	1.07E-02	rs10035105
ENSG00000005381	*MPO*	4	1.07E-02	rs1911822
ENSG00000100652	*SLC10A1*	2	1.07E-02	rs6894851
ENSG00000107242	*PIP5K1B*	2	1.07E-02	rs10076681
ENSG00000136381	*IREB2*	4	1.07E-02	rs1508547
ENSG00000242612	*DECR2*	2	1.07E-02	rs10073450
ENSG00000072210	*ALDH3A2*	2	1.08E-02	rs10079950
ENSG00000117448	*AKR1A1*	3	1.08E-02	rs6872378
ENSG00000160209	*PDXK*	4	1.08E-02	rs1703052
ENSG00000165264	*NDUFB6*	3	1.08E-02	rs4866039
ENSG00000118363	*SPCS2*	4	1.08E-02	rs4288117
ENSG00000145217	*SLC26A1*	2	1.09E-02	rs1911845
ENSG00000125454	*SLC25A19*	4	1.09E-02	rs4288117
ENSG00000115944	*COX7A2L*	3	1.09E-02	rs7734813
ENSG00000182870	*GALNT9*	2	1.10E-02	rs12652510
ENSG00000078070	*MCCC1*	2	1.10E-02	rs13357704
ENSG00000100889	*PCK2*	4	1.10E-02	rs11738280
ENSG00000148384	*INPP5E*	1	1.10E-02	rs10073450
ENSG00000157881	*PANK4*	4	1.10E-02	rs6869352
ENSG00000112299	*VNN1*	2	1.11E-02	rs13188919
ENSG00000140263	*SORD*	2	1.11E-02	rs6872378
ENSG00000137261	*KIAA0319*	2	1.11E-02	rs10078676
ENSG00000160870	*CYP3A7*	2	1.11E-02	rs7708358
ENSG00000114956	*DGUOK*	4	1.11E-02	rs1508547
ENSG00000138735	*PDE5A*	2	1.12E-02	rs1876603
ENSG00000107798	*LIPA*	4	1.12E-02	rs73050988
ENSG00000058866	*DGKG*	2	1.12E-02	rs4392624
ENSG00000128683	*GAD1*	2	1.12E-02	rs13184938
ENSG00000108576	*SLC6A4*	4	1.12E-02	rs6884961
ENSG00000113273	*ARSB*	4	1.12E-02	rs2434785
ENSG00000143797	*MBOAT2*	2	1.12E-02	rs2136102
ENSG00000102144	*PGK1*	3	1.13E-02	rs4866148
ENSG00000136169	*SETDB2*	2	1.13E-02	rs11745155
ENSG00000070915	*SLC12A3*	2	1.13E-02	rs6877123
ENSG00000112992	*NNT*	2	1.14E-02	rs16888249
ENSG00000147576	*ADHFE1*	2	1.14E-02	rs13178644
ENSG00000137491	*SLCO2B1*	2	1.14E-02	rs67911776
ENSG00000064601	*CTSA*	4	1.14E-02	rs9292895
ENSG00000241360	*PDXP*	2	1.14E-02	rs4554223
ENSG00000119899	*SLC17A5*	1	1.14E-02	rs1346543
ENSG00000167186	*COQ7*	2	1.14E-02	rs919344
ENSG00000108242	*CYP2C18*	2	1.14E-02	rs7713439
ENSG00000122884	*P4HA1*	4	1.14E-02	rs7734813
ENSG00000127415	*IDUA*	2	1.14E-02	rs10066903
ENSG00000153976	*HS3ST3A1*	2	1.15E-02	rs1911822
ENSG00000128050	*PAICS*	4	1.16E-02	rs7720726
ENSG00000131480	*AOC2*	2	1.16E-02	rs13357704
ENSG00000049860	*HEXB*	4	1.16E-02	rs7708358
ENSG00000205268	*PDE7A*	2	1.16E-02	rs1347523
ENSG00000131183	*SLC34A1*	2	1.16E-02	rs164448
ENSG00000162694	*EXTL2*	4	1.16E-02	rs6887118
ENSG00000070669	*ASNS*	2	1.17E-02	rs4866148

ENSG00000131473	*ACLY*	4	1.17E-02	rs1155040
ENSG00000139531	*SUOX*	4	1.17E-02	rs1508547
ENSG00000112303	*VNN2*	2	1.18E-02	rs11738280
ENSG00000087085	*ACHE*	2	1.18E-02	rs6884961
ENSG00000177628	*GBA*	4	1.18E-02	rs1030479
ENSG00000125877	*ITPA*	3	1.18E-02	rs1909117
ENSG00000117411	*B4GALT2*	1	1.18E-02	rs9986203
ENSG00000120697	*ALG5*	2	1.18E-02	rs4554223
ENSG00000004468	*CD38*	2	1.19E-02	rs6888503
ENSG00000157593	*SLC35B2*	4	1.19E-02	rs10041937
ENSG00000163283	*ALPP*	2	1.19E-02	rs75841406
ENSG00000171302	*CANT1*	4	1.19E-02	rs11738280
ENSG00000101187	*SLCO4A1*	2	1.19E-02	rs10078676
ENSG00000005471	*ABCB4*	2	1.19E-02	rs11740264
ENSG00000102125	*TAZ*	4	1.20E-02	rs4866150
ENSG00000231852	*NA*	2	1.20E-02	rs11959190
ENSG00000127184	*COX7C*	3	1.20E-02	rs7708358
ENSG00000067113	*PLPP1*	2	1.20E-02	rs1703047
ENSG00000144741	*SLC25A26*	4	1.20E-02	rs2974586
ENSG00000117592	*PRDX6*	3	1.21E-02	rs10066903
ENSG00000161013	*MGAT4B*	4	1.21E-02	rs10035105
ENSG00000164919	*COX6C*	3	1.21E-02	rs6894851
ENSG00000175567	*UCP2*	4	1.22E-02	rs13164886
ENSG00000159063	*ALG8*	4	1.22E-02	rs2136125
ENSG00000162066	*AMDHD2*	2	1.22E-02	rs10473357
ENSG00000147408	*CSGALNACT1*	2	1.22E-02	rs73762480
ENSG00000099377	*HSD3B7*	1	1.22E-02	rs11739635
ENSG00000114021	*NIT2*	3	1.22E-02	rs1498103
ENSG00000106049	*HIBADH*	4	1.22E-02	rs1498103
ENSG00000141504	*SAT2*	3	1.23E-02	rs1909117
ENSG00000114902	*SPCS1*	4	1.23E-02	rs13357704
ENSG00000104524	*PYCR3*	4	1.23E-02	rs1703047
ENSG00000127472	*PLA2G5*	2	1.23E-02	rs12514159
ENSG00000001036	*FUCA2*	4	1.24E-02	rs13170493
ENSG00000124357	*NAGK*	2	1.24E-02	rs1911845
ENSG00000178445	*GLDC*	2	1.24E-02	rs1911845
ENSG00000198814	*GK*	2	1.24E-02	rs13360869
ENSG00000167280	*ENGASE*	2	1.25E-02	rs2115342
ENSG00000102780	*DGKH*	2	1.26E-02	rs11738280
ENSG00000025708	*TYMP*	2	1.26E-02	rs10073450
ENSG00000166340	*TPP1*	4	1.26E-02	rs1521023
ENSG00000160326	*SLC2A6*	2	1.26E-02	rs73056742
ENSG00000130707	*ASS1*	4	1.26E-02	rs62351320
ENSG00000149150	*SLC43A1*	4	1.26E-02	rs13170493
ENSG00000057252	*SOAT1*	4	1.26E-02	rs11952071
ENSG00000080819	*CPOX*	2	1.26E-02	rs55724955
ENSG00000101911	*PRPS2*	4	1.26E-02	rs7736515
ENSG00000228716	*DHFR*	4	1.26E-02	rs919344
ENSG00000129187	*DCTD*	4	1.27E-02	rs10056397
ENSG00000148377	*IDI2*	4	1.27E-02	rs2471148
ENSG00000169710	*FASN*	4	1.27E-02	rs4866190
ENSG00000203797	*DDO*	2	1.27E-02	rs1521026
ENSG00000124140	*SLC12A5*	2	1.28E-02	rs73050988
ENSG00000125255	*SLC10A2*	4	1.28E-02	rs2060533
ENSG00000204386	*NEU1*	4	1.28E-02	rs4084799
ENSG00000141349	*G6PC3*	4	1.28E-02	rs16888249
ENSG00000167792	*NDUFV1*	3	1.29E-02	rs2115342
ENSG00000138079	*SLC3A1*	4	1.29E-02	rs4866039

ENSG00000176340	*COX8A*	1	1.29E-02	rs16888249
ENSG00000164904	*ALDH7A1*	2	1.30E-02	rs7720726
ENSG00000128274	*A4GALT*	2	1.30E-02	rs13360869
ENSG00000160216	*AGPAT3*	4	1.30E-02	rs13183491
ENSG00000119537	*KDSR*	4	1.30E-02	rs7720726
ENSG00000130066	*SAT1*	4	1.32E-02	rs6887953
ENSG00000136521	*NDUFB5*	4	1.33E-02	rs10473357
ENSG00000185808	*PIGP*	4	1.33E-02	rs11739635
ENSG00000166908	*PIP4K2C*	2	1.33E-02	rs2471114
ENSG00000165841	*CYP2C19*	2	1.33E-02	rs17222830
ENSG00000138061	*CYP1B1*	2	1.33E-02	rs12659684
ENSG00000076351	*SLC46A1*	4	1.33E-02	rs1508547
ENSG00000157350	*ST3GAL2*	4	1.34E-02	rs10941391
ENSG00000117308	*GALE*	4	1.34E-02	rs7736515
ENSG00000176974	*SHMT1*	4	1.34E-02	rs13174782
ENSG00000166825	*ANPEP*	2	1.35E-02	rs2974591
ENSG00000115840	*SLC25A12*	4	1.36E-02	rs4288117
ENSG00000115902	*SLC1A4*	4	1.36E-02	rs4401574
ENSG00000117461	*PIK3R3*	2	1.36E-02	rs4400126
ENSG00000130313	*PGLS*	1	1.36E-02	rs16888249
ENSG00000146587	*RBAK*	2	1.37E-02	rs11952071
ENSG00000178537	*SLC25A20*	4	1.37E-02	rs28648852
ENSG00000105379	*ETFB*	1	1.37E-02	rs11952071
ENSG00000109814	*UGDH*	2	1.38E-02	rs4422533
ENSG00000131143	*COX4I1*	1	1.39E-02	rs1508547
ENSG00000114480	*GBE1*	2	1.39E-02	rs12153263
ENSG00000101333	*PLCB4*	2	1.39E-02	rs13178644
ENSG00000160688	*FLAD1*	2	1.39E-02	rs7734813
ENSG00000158850	*B4GALT3*	2	1.40E-02	rs35738662
ENSG00000132915	*PDE6A*	2	1.40E-02	rs55724955
ENSG00000130649	*CYP2E1*	2	1.41E-02	rs1909117
ENSG00000197375	*SLC22A5*	4	1.41E-02	rs13170493
ENSG00000101986	*ABCD1*	2	1.41E-02	rs11744487
ENSG00000162836	*ACP6*	2	1.42E-02	rs10078346
ENSG00000142319	*SLC6A3*	2	1.42E-02	rs7736515
ENSG00000038274	*MAT2B*	4	1.43E-02	rs10045245
ENSG00000162882	*HAAO*	4	1.43E-02	rs1435971
ENSG00000004455	*AK2*	4	1.43E-02	rs10066903
ENSG00000165644	*COMTD1*	1	1.44E-02	rs10066903
ENSG00000102030	*NAA10*	2	1.44E-02	rs9986203
ENSG00000070614	*NDST1*	4	1.44E-02	rs11952071
ENSG00000197506	*SLC28A3*	2	1.44E-02	rs6877123
ENSG00000111674	*ENO2*	4	1.45E-02	rs1549653
ENSG00000112541	*PDE10A*	2	1.45E-02	rs189492
ENSG00000143774	*GUK1*	1	1.45E-02	rs16888249
ENSG00000135069	*PSAT1*	2	1.45E-02	rs6451845
ENSG00000125166	*GOT2*	3	1.45E-02	rs10065518
ENSG00000149091	*DGKZ*	2	1.46E-02	rs10066903
ENSG00000182199	*SHMT2*	2	1.46E-02	rs6887118
ENSG00000160226	*C21orf2*	2	1.46E-02	rs73050988
ENSG00000010379	*SLC6A13*	2	1.46E-02	rs75841406
ENSG00000198431	*TXNRD1*	2	1.46E-02	rs11738280
ENSG00000116906	*GNPAT*	4	1.47E-02	rs10066903
ENSG00000177191	*B3GNT8*	2	1.47E-02	rs11744487
ENSG00000118705	*RPN2*	4	1.47E-02	rs73056742
ENSG00000196547	*MAN2A2*	4	1.48E-02	rs2115342
ENSG00000111666	*CHPT1*	2	1.48E-02	rs17839229
ENSG00000235863	*B3GALT4*	4	1.48E-02	rs10078346

ENSG00000186792	*HYAL3*	2	1.48E-02	rs72745083
ENSG00000130005	*GAMT*	1	1.48E-02	rs10045245
ENSG00000153936	*HS2ST1*	2	1.49E-02	rs13360869
ENSG00000104763	*ASAH1*	4	1.49E-02	rs35773719
ENSG00000166136	*NDUFB8*	2	1.50E-02	rs2202798
ENSG00000127884	*ECHS1*	4	1.51E-02	rs2892442
ENSG00000177646	*ACAD9*	4	1.51E-02	rs10060069
ENSG00000151498	*ACAD8*	4	1.51E-02	rs55724955
ENSG00000103064	*SLC7A6*	2	1.51E-02	rs4866039
ENSG00000178921	*PFAS*	4	1.51E-02	rs7734813
ENSG00000055609	*KMT2C*	4	1.52E-02	rs2471154
ENSG00000172890	*NADSYN1*	2	1.52E-02	rs17222830
ENSG00000174669	*SLC29A2*	4	1.53E-02	rs4554223
ENSG00000158571	*PFKFB1*	2	1.54E-02	rs1521023
ENSG00000167780	*SOAT2*	2	1.54E-02	rs7720726
ENSG00000181090	*EHMT1*	2	1.55E-02	rs1396340
ENSG00000171989	*LDHAL6B*	2	1.55E-02	rs11952071
ENSG00000125779	*PANK2*	4	1.55E-02	rs71610668
ENSG00000100714	*MTHFD1*	4	1.55E-02	rs10069647
ENSG00000017483	*SLC38A5*	4	1.55E-02	rs73056742
ENSG00000112309	*B3GAT2*	3	1.55E-02	rs1521026
ENSG00000197165	*SULT1A2*	2	1.56E-02	rs10066903
ENSG00000133056	*PIK3C2B*	4	1.56E-02	rs10068995
ENSG00000196433	*ASMT*	2	1.56E-02	rs6883590
ENSG00000166224	*SGPL1*	4	1.56E-02	rs7734813
ENSG00000108474	*PIGL*	4	1.56E-02	rs13360869
ENSG00000117479	*SLC19A2*	4	1.57E-02	rs1508547
ENSG00000182054	*IDH2*	4	1.57E-02	rs164448
ENSG00000157064	*NMNAT2*	2	1.57E-02	rs13153197
ENSG00000147647	*DPYS*	2	1.57E-02	rs13190303
ENSG00000128242	*GAL3ST1*	2	1.57E-02	rs11739635
ENSG00000183955	*KMT5A*	4	1.58E-02	rs16888249
ENSG00000111775	*COX6A1*	4	1.58E-02	rs4288117
ENSG00000198276	*UCKL1*	1	1.58E-02	rs10473307
ENSG00000116157	*GPX7*	2	1.59E-02	rs35738662
ENSG00000068366	*ACSL4*	4	1.60E-02	rs10045245
ENSG00000143379	*SETDB1*	2	1.60E-02	rs1911845
ENSG00000168918	*INPP5D*	2	1.61E-02	rs13170493
ENSG00000059573	*ALDH18A1*	4	1.61E-02	rs6887953
ENSG00000063601	*MTMR1*	4	1.61E-02	rs2940461
ENSG00000159231	*CBR3*	2	1.61E-02	rs1521026
ENSG00000211445	*GPX3*	2	1.62E-02	rs13188919
ENSG00000067225	*PKM*	4	1.62E-02	rs59199016
ENSG00000230359	*TPI1P2*	3	1.62E-02	rs1909117
ENSG00000085662	*AKR1B1*	4	1.64E-02	rs13360869
ENSG00000186111	*PIP5K1C*	2	1.65E-02	rs16888249
ENSG00000198650	*TAT*	2	1.65E-02	rs12109816
ENSG00000005469	*CROT*	2	1.65E-02	rs7734813
ENSG00000065518	*NDUFB4*	3	1.65E-02	rs1521023
ENSG00000117620	*SLC35A3*	4	1.66E-02	rs61441877
ENSG00000196620	*UGT2B15*	2	1.66E-02	rs6877123
ENSG00000008394	*MGST1*	2	1.66E-02	rs1549653
ENSG00000115275	*MOGS*	4	1.66E-02	rs1347523
ENSG00000047457	*CP*	2	1.67E-02	rs10045245
ENSG00000160191	*PDE9A*	4	1.67E-02	rs6863000
ENSG00000113448	*PDE4D*	2	1.68E-02	rs10069647
ENSG00000054983	*GALC*	4	1.68E-02	rs7716704
ENSG00000165996	*HACD1*	2	1.68E-02	rs34294319

ENSG00000164258	*NDUFS4*	3	1.68E-02	rs7734813
ENSG00000069943	*PIGB*	2	1.69E-02	rs62351320
ENSG00000163754	*GYG1*	2	1.70E-02	rs7705221
ENSG00000174607	*UGT8*	2	1.70E-02	rs16888249
ENSG00000164978	*NUDT2*	2	1.70E-02	rs55822426
ENSG00000071073	*MGAT4A*	2	1.71E-02	rs6877123
ENSG00000143252	*SDHC*	4	1.71E-02	rs1549653
ENSG00000129467	*ADCY4*	2	1.71E-02	rs1911845
ENSG00000149782	*PLCB3*	4	1.72E-02	rs10472421
ENSG00000164742	*ADCY1*	2	1.72E-02	rs11744487
ENSG00000111669	*TPI1*	3	1.72E-02	rs7708358
ENSG00000095303	*PTGS1*	2	1.72E-02	rs4422533
ENSG00000013392	*RWDD2A*	4	1.73E-02	rs6869124
ENSG00000158470	*B4GALT5*	4	1.73E-02	rs1876591
ENSG00000184254	*ALDH1A3*	4	1.73E-02	rs16888249
ENSG00000167900	*TK1*	2	1.73E-02	rs12110309
ENSG00000117143	*UAP1*	4	1.73E-02	rs73056763
ENSG00000123213	*NLN*	4	1.74E-02	rs10069647
ENSG00000116771	*AGMAT*	4	1.74E-02	rs10068995
ENSG00000161714	*PLCD3*	4	1.74E-02	rs16885636
ENSG00000169359	*SLC33A1*	2	1.75E-02	rs4400126
ENSG00000197496	*SLC2A10*	2	1.75E-02	rs1876591
ENSG00000164707	*SLC13A4*	2	1.76E-02	rs6883590
ENSG00000163399	*ATP1A1*	4	1.76E-02	rs1911845
ENSG00000090020	*SLC9A1*	2	1.77E-02	rs7720726
ENSG00000116704	*SLC35D1*	4	1.77E-02	rs1508547
ENSG00000143641	*GALNT2*	2	1.77E-02	rs9687957
ENSG00000133256	*PDE6B*	4	1.78E-02	rs13360869
ENSG00000158467	*AHCYL2*	2	1.79E-02	rs55724955
ENSG00000173085	*COQ2*	4	1.79E-02	rs7720726
ENSG00000214160	*ALG3*	4	1.80E-02	rs1909117
ENSG00000143156	*NME7*	4	1.81E-02	rs10076681
ENSG00000115419	*GLS*	4	1.81E-02	rs11740264
ENSG00000197888	*UGT2B17*	2	1.82E-02	rs6877123
ENSG00000185015	*CA13*	2	1.82E-02	rs1703047
ENSG00000155380	*SLC16A1*	2	1.82E-02	rs10066903
ENSG00000126522	*ASL*	4	1.82E-02	rs1498103
ENSG00000166548	*TK2*	2	1.83E-02	rs2974591
ENSG00000135821	*GLUL*	2	1.83E-02	rs11740264
ENSG00000183048	*SLC25A10*	2	1.84E-02	rs7721392
ENSG00000026652	*AGPAT4*	2	1.85E-02	rs10066903
ENSG00000104522	*TSTA3*	4	1.85E-02	rs6887953
ENSG00000141506	*PIK3R5*	2	1.86E-02	rs10045245
ENSG00000087157	*PGS1*	4	1.86E-02	rs7708358
ENSG00000138193	*PLCE1*	2	1.87E-02	rs59277639
ENSG00000188690	*UROS*	4	1.87E-02	rs7734813
ENSG00000107614	*TRDMT1*	4	1.88E-02	rs3112489
ENSG00000100994	*PYGB*	4	1.88E-02	rs10473307
ENSG00000168092	*PAFAH1B2*	4	1.90E-02	rs34294319
ENSG00000131781	*FMO5*	2	1.90E-02	rs11741365
ENSG00000127125	*PPCS*	2	1.91E-02	rs7734813
ENSG00000168393	*DTYMK*	1	1.91E-02	rs1703047
ENSG00000147416	*ATP6V1B2*	2	1.92E-02	rs57202385
ENSG00000146411	*SLC2A12*	2	1.92E-02	rs72745083
ENSG00000144136	*SLC20A1*	2	1.93E-02	rs12652510
ENSG00000143149	*ALDH9A1*	4	1.93E-02	rs1508547
ENSG00000106392	*C1GALT1*	2	1.93E-02	rs7705221
ENSG00000179085	*DPM3*	1	1.93E-02	rs4866039

ENSG00000099795	*NDUFB7*	1	1.94E-02	rs12110309
ENSG00000159228	*CBR1*	2	1.95E-02	rs10941842
ENSG00000174915	*PTDSS2*	2	1.95E-02	rs17839229
ENSG00000148832	*PAOX*	2	1.96E-02	rs1911845
ENSG00000128524	*ATP6V1F*	4	1.96E-02	rs6884683
ENSG00000111728	*ST8SIA1*	4	1.96E-02	rs7720726
ENSG00000142046	*TMEM91*	4	1.97E-02	rs6863000
ENSG00000060971	*ACAA1*	2	1.97E-02	rs10066903
ENSG00000138029	*HADHB*	3	1.97E-02	rs55822426
ENSG00000169020	*ATP5ME*	3	1.97E-02	rs12110309
ENSG00000139684	*ESD*	3	1.98E-02	rs10473357
ENSG00000063854	*HAGH*	4	1.98E-02	rs1521026
ENSG00000088451	*TGDS*	4	1.98E-02	rs13170493
ENSG00000171862	*PTEN*	4	1.99E-02	rs11952071
ENSG00000138413	*IDH1*	4	2.00E-02	rs6870586
ENSG00000172340	*SUCLG2*	2	2.00E-02	rs1521023
ENSG00000147155	*EBP*	4	2.00E-02	rs72745083
ENSG00000133805	*AMPD3*	2	2.01E-02	rs1521026
ENSG00000101473	*ACOT8*	4	2.01E-02	rs16888249
ENSG00000100292	*HMOX1*	2	2.02E-02	rs6884961
ENSG00000101846	*STS*	2	2.03E-02	rs10066903
ENSG00000105647	*PIK3R2*	4	2.03E-02	rs1521026
ENSG00000132570	*PCBD2*	4	2.03E-02	rs2471154
ENSG00000240038	*AMY2B*	4	2.04E-02	rs2136102
ENSG00000166743	*ACSM1*	2	2.04E-02	rs1549653
ENSG00000185013	*NT5C1B*	2	2.04E-02	rs4866039
ENSG00000123453	*SARDH*	2	2.05E-02	rs2974586
ENSG00000164879	*CA3*	2	2.05E-02	rs13183491
ENSG00000127540	*UQCR11*	1	2.05E-02	rs73056793
ENSG00000170266	*GLB1*	2	2.05E-02	rs7713439
ENSG00000120915	*EPHX2*	2	2.06E-02	rs1911845
ENSG00000169169	*CPT1C*	2	2.08E-02	rs13183491
ENSG00000130414	*NDUFA10*	2	2.08E-02	rs2434785
ENSG00000240857	*RDH14*	4	2.08E-02	rs1521026
ENSG00000141959	*PFKL*	1	2.08E-02	rs1549653
ENSG00000109667	*SLC2A9*	2	2.09E-02	rs994482
ENSG00000165282	*PIGO*	4	2.09E-02	rs57202385
ENSG00000129244	*ATP1B2*	2	2.10E-02	rs1521026
ENSG00000115616	*SLC9A2*	2	2.10E-02	rs11738280
ENSG00000160868	*CYP3A4*	2	2.11E-02	rs34294319
ENSG00000155097	*ATP6V1C1*	2	2.11E-02	rs1521026
ENSG00000111012	*CYP27B1*	2	2.11E-02	rs61441877
ENSG00000118596	*SLC16A7*	4	2.12E-02	rs6869124
ENSG00000133027	*PEMT*	1	2.12E-02	rs10066903
ENSG00000131730	*CKMT2*	2	2.13E-02	rs12517892
ENSG00000101638	*ST8SIA5*	2	2.13E-02	rs2940461
ENSG00000167996	*FTH1*	2	2.13E-02	rs10941391
ENSG00000131459	*GFPT2*	2	2.13E-02	rs13360869
ENSG00000081181	*ARG2*	4	2.13E-02	rs10066903
ENSG00000101438	*SLC32A1*	2	2.15E-02	rs4866190
ENSG00000197448	*GSTK1*	4	2.15E-02	rs2892442
ENSG00000065154	*OAT*	2	2.15E-02	rs1396340
ENSG00000090266	*NDUFB2*	3	2.15E-02	rs6888503
ENSG00000213316	*LTC4S*	2	2.15E-02	rs12110309
ENSG00000100024	*UPB1*	2	2.15E-02	rs28648852
ENSG00000068383	*INPP5A*	2	2.16E-02	rs4422533
ENSG00000128268	*MGAT3*	2	2.16E-02	rs11745155
ENSG00000198805	*PNP*	4	2.17E-02	rs73050988

ENSG00000178127	*NDUFV2*	3	2.17E-02	rs1030479
ENSG00000184983	*NDUFA6*	2	2.18E-02	rs994482
ENSG00000136371	*MTHFS*	2	2.18E-02	rs57202385
ENSG00000101670	*LIPG*	2	2.18E-02	rs58017518
ENSG00000147119	*CHST7*	2	2.18E-02	rs2202798
ENSG00000108786	*HSD17B1*	4	2.19E-02	rs4588601
ENSG00000156966	*B3GNT7*	4	2.19E-02	rs3112489
ENSG00000184076	*UQCR10*	3	2.20E-02	rs1472892
ENSG00000143811	*PYCR2*	2	2.20E-02	rs13174782
ENSG00000157326	*DHRS4*	4	2.21E-02	rs62351320
ENSG00000114805	*PLCH1*	2	2.23E-02	rs1508547
ENSG00000134013	*LOXL2*	2	2.23E-02	rs4404681
ENSG00000159199	*ATP5MC1*	4	2.24E-02	rs7713439
ENSG00000173786	*CNP*	2	2.24E-02	rs6884961
ENSG00000108839	*ALOX12*	4	2.24E-02	rs1346543
ENSG00000239672	*NME1*	4	2.25E-02	rs7736515
ENSG00000131873	*CHSY1*	1	2.25E-02	rs9687957
ENSG00000158825	*CDA*	2	2.25E-02	rs4866150
ENSG00000139547	*RDH16*	4	2.27E-02	rs3112489
ENSG00000183648	*NDUFB1*	3	2.27E-02	rs73050988
ENSG00000214013	*GANC*	2	2.28E-02	rs55822426
ENSG00000145545	*SRD5A1*	4	2.28E-02	rs12652510
ENSG00000061918	*GUCY1B1*	2	2.28E-02	rs9292895
ENSG00000168237	*GLYCTK*	2	2.29E-02	rs57878094
ENSG00000243678	*NME2*	4	2.30E-02	rs1508547
ENSG00000114573	*ATP6V1A*	4	2.31E-02	rs1508547
ENSG00000100504	*PYGL*	2	2.31E-02	rs62355216
ENSG00000100522	*GNPNAT1*	4	2.32E-02	rs6884683
ENSG00000111144	*LTA4H*	4	2.32E-02	rs11740264
ENSG00000150867	*PIP4K2A*	4	2.33E-02	rs7736515
ENSG00000159720	*ATP6V0D1*	2	2.34E-02	rs10472421
ENSG00000111732	*AICDA*	2	2.35E-02	rs7705221
ENSG00000002587	*HS3ST1*	2	2.36E-02	rs1909117
ENSG00000198964	*SGMS1*	2	2.36E-02	rs4084799
ENSG00000111261	*MANSC1*	2	2.37E-02	rs73056742
ENSG00000117305	*HMGCL*	4	2.37E-02	rs10041937
ENSG00000198743	*SLC5A3*	4	2.39E-02	rs1508547
ENSG00000104885	*DOT1L*	1	2.39E-02	rs6883590
ENSG00000155252	*PI4K2A*	2	2.40E-02	rs10066903
ENSG00000155465	*SLC7A7*	2	2.40E-02	rs6451845
ENSG00000140990	*NDUFB10*	1	2.40E-02	rs12110309
ENSG00000165704	*HPRT1*	4	2.40E-02	rs10065518
ENSG00000109586	*GALNT7*	4	2.41E-02	rs9986203
ENSG00000172955	*ADH6*	2	2.42E-02	rs1508547
ENSG00000151552	*QDPR*	2	2.42E-02	rs12110309
ENSG00000072778	*ACADVL*	4	2.46E-02	rs1155040
ENSG00000104325	*DECR1*	4	2.46E-02	rs7708358
ENSG00000007541	*PIGQ*	1	2.46E-02	rs3112489
ENSG00000179918	*SEPHS2*	4	2.47E-02	rs10066903
ENSG00000164116	*GUCY1A1*	2	2.47E-02	rs6884961
ENSG00000099810	*MTAP*	4	2.48E-02	rs11741365
ENSG00000169826	*CSGALNACT2*	4	2.49E-02	rs6887953
ENSG00000119711	*ALDH6A1*	2	2.52E-02	rs7736515
ENSG00000095464	*PDE6C*	2	2.52E-02	rs6870586
ENSG00000170340	*B3GNT2*	4	2.55E-02	rs11952071
ENSG00000134716	*CYP2J2*	2	2.57E-02	rs34294319
ENSG00000119689	*DLST*	4	2.58E-02	rs10041937
ENSG00000123454	*DBH*	2	2.60E-02	rs1703047

ENSG00000033867	*SLC4A7*	4	2.61E-02	rs994482
ENSG00000120137	*PANK3*	4	2.62E-02	rs11738280
ENSG00000171234	*UGT2B7*	2	2.62E-02	rs4422533
ENSG00000178741	*COX5A*	4	2.63E-02	rs1508547
ENSG00000149485	*FADS1*	4	2.63E-02	rs13170493
ENSG00000086062	*B4GALT1*	4	2.64E-02	rs11744487
ENSG00000033170	*FUT8*	2	2.64E-02	rs16888249
ENSG00000129596	*CDO1*	2	2.65E-02	rs13357704
ENSG00000100678	*SLC8A3*	2	2.65E-02	rs35738662
ENSG00000078269	*SYNJ2*	2	2.66E-02	rs55822426
ENSG00000120053	*GOT1*	4	2.66E-02	rs1911822
ENSG00000136720	*HS6ST1*	1	2.67E-02	rs7734813
ENSG00000240891	*PLCXD2*	2	2.67E-02	rs62355178
ENSG00000197208	*SLC22A4*	2	2.68E-02	rs6863000
ENSG00000105650	*PDE4C*	2	2.68E-02	rs35773719
ENSG00000156467	*UQCRB*	2	2.73E-02	rs13357704
ENSG00000159921	*GNE*	2	2.74E-02	rs6451845
ENSG00000167306	*MYO5B*	4	2.75E-02	rs10065518
ENSG00000014257	*ACPP*	2	2.76E-02	rs12658322
ENSG00000072506	*HSD17B10*	4	2.76E-02	rs7736515
ENSG00000177239	*MAN1B1*	4	2.76E-02	rs6870271
ENSG00000070731	*ST6GALNAC2*	2	2.77E-02	rs12659684
ENSG00000143627	*PKLR*	2	2.77E-02	rs1396340
ENSG00000140612	*SEC11A*	4	2.77E-02	rs55822426
ENSG00000163082	*SGPP2*	2	2.77E-02	rs1472892
ENSG00000171298	*GAA*	2	2.78E-02	rs10056397
ENSG00000204371	*EHMT2*	2	2.78E-02	rs1911845
ENSG00000135002	*RFK*	4	2.80E-02	rs11748566
ENSG00000125458	*NT5C*	1	2.81E-02	rs10066903
ENSG00000103150	*MLYCD*	4	2.81E-02	rs2471154
ENSG00000137106	*GRHPR*	4	2.83E-02	rs7736515
ENSG00000148334	*PTGES2*	4	2.84E-02	rs16888249
ENSG00000122912	*SLC25A16*	4	2.85E-02	rs61441877
ENSG00000157353	*FUK*	2	2.89E-02	rs57878094
ENSG00000111885	*MAN1A1*	4	2.89E-02	rs4400126
ENSG00000145675	*PIK3R1*	4	2.90E-02	rs1508547
ENSG00000178814	*OPLAH*	2	2.90E-02	rs13190303
ENSG00000067829	*IDH3G*	2	2.91E-02	rs1703049
ENSG00000163964	*PIGX*	3	2.91E-02	rs10073450
ENSG00000102078	*SLC25A14*	2	2.92E-02	rs1540751
ENSG00000135437	*RDH5*	4	2.92E-02	rs11738280
ENSG00000138031	*ADCY3*	2	2.92E-02	rs1549653
ENSG00000160285	*LSS*	4	2.94E-02	rs189492
ENSG00000115850	*LCT*	2	2.95E-02	rs1911822
ENSG00000185633	*NDUFA4L2*	2	2.95E-02	rs11741365
ENSG00000072042	*RDH11*	4	2.97E-02	rs994482
ENSG00000183044	*ABAT*	2	2.97E-02	rs1521026
ENSG00000151348	*EXT2*	2	2.97E-02	rs1346543
ENSG00000170634	*ACYP2*	4	3.02E-02	rs10045245
ENSG00000170482	*SLC23A1*	2	3.02E-02	rs10073450
ENSG00000110721	*CHKA*	2	3.05E-02	rs4422533
ENSG00000111371	*SLC38A1*	4	3.05E-02	rs55749186
ENSG00000141934	*PLPP2*	2	3.05E-02	rs1521026
ENSG00000018510	*AGPS*	2	3.07E-02	rs10074201
ENSG00000130958	*SLC35D2*	2	3.07E-02	rs10045245
ENSG00000142102	*PGGHG*	4	3.08E-02	rs2063227
ENSG00000158516	*CPA2*	2	3.08E-02	rs12110309
ENSG00000179761	*PIPOX*	4	3.10E-02	rs1521023

ENSG00000165029	*ABCA1*	2	3.10E-02	rs1703047
ENSG00000140090	*SLC24A4*	2	3.10E-02	rs10060069
ENSG00000170950	*PGK2*	2	3.11E-02	rs2254473
ENSG00000198380	*GFPT1*	4	3.12E-02	rs10066903
ENSG00000121039	*RDH10*	2	3.13E-02	rs55749186
ENSG00000138777	*PPA2*	4	3.13E-02	rs10060069
ENSG00000141485	*SLC13A5*	2	3.15E-02	rs10472421
ENSG00000124172	*ATP5F1E*	4	3.17E-02	rs2471154
ENSG00000151229	*SLC2A13*	2	3.18E-02	rs10056397
ENSG00000198951	*NAGA*	4	3.19E-02	rs10066903
ENSG00000143727	*ACP1*	2	3.19E-02	rs1521026
ENSG00000137841	*PLCB2*	2	3.20E-02	rs745105
ENSG00000186529	*CYP4F3*	2	3.20E-02	rs1703047
ENSG00000167588	*GPD1*	2	3.22E-02	rs10073450
ENSG00000164414	*SLC35A1*	2	3.22E-02	rs1347523
ENSG00000089250	*NOS1*	2	3.22E-02	rs55822426
ENSG00000007168	*PAFAH1B1*	4	3.23E-02	rs10045245
ENSG00000179477	*ALOX12B*	2	3.26E-02	rs13357704
ENSG00000167863	*ATP5PD*	3	3.27E-02	rs73050988
ENSG00000073578	*SDHA*	2	3.27E-02	rs1909117
ENSG00000147123	*NDUFB11*	4	3.28E-02	rs7721392
ENSG00000132330	*SCLY*	4	3.28E-02	rs35837974
ENSG00000160439	*RDH13*	4	3.29E-02	rs7736515
ENSG00000158525	*CPA5*	2	3.30E-02	rs11739635
ENSG00000141012	*GALNS*	4	3.31E-02	rs13360869
ENSG00000197586	*ENTPD6*	4	3.33E-02	rs11956407
ENSG00000165646	*SLC18A2*	4	3.34E-02	rs1521026
ENSG00000089057	*SLC23A2*	2	3.35E-02	rs73056742
ENSG00000115525	*ST3GAL5*	4	3.37E-02	rs1911822
ENSG00000115556	*PLCD4*	2	3.37E-02	rs11738280
ENSG00000151116	*UEVLD*	4	3.38E-02	rs16888249
ENSG00000176022	*B3GALT6*	1	3.38E-02	rs9687957
ENSG00000139133	*ALG10*	4	3.39E-02	rs10035105
ENSG00000184005	*ST6GALNAC3*	2	3.42E-02	rs6887953
ENSG00000140057	*AK7*	2	3.43E-02	rs745105
ENSG00000059804	*SLC2A3*	4	3.44E-02	rs1549653
ENSG00000073969	*NSF*	2	3.46E-02	rs10066903
ENSG00000176597	*B3GNT5*	2	3.46E-02	rs7716704
ENSG00000121879	*PIK3CA*	4	3.46E-02	rs11738280
ENSG00000131446	*MGAT1*	4	3.46E-02	rs1521026
ENSG00000213619	*NDUFS3*	4	3.46E-02	rs1352199
ENSG00000143199	*ADCY10*	2	3.47E-02	rs10056397
ENSG00000033011	*ALG1*	4	3.48E-02	rs7721392
ENSG00000184381	*PLA2G6*	4	3.50E-02	rs1472892
ENSG00000181830	*SLC35C1*	4	3.51E-02	rs6884961
ENSG00000136872	*ALDOB*	2	3.51E-02	rs1508547
ENSG00000143153	*ATP1B1*	2	3.52E-02	rs1549653
ENSG00000136960	*ENPP2*	2	3.55E-02	rs75841406
ENSG00000181523	*SGSH*	4	3.55E-02	rs35738662
ENSG00000111271	*ACAD10*	2	3.55E-02	rs2202798
ENSG00000118276	*B4GALT6*	4	3.56E-02	rs11738280
ENSG00000181045	*SLC26A11*	4	3.59E-02	rs1521023
ENSG00000206527	*HACD2*	4	3.60E-02	rs10035105
ENSG00000065833	*ME1*	2	3.62E-02	rs6872378
ENSG00000115159	*GPD2*	4	3.64E-02	rs11738280
ENSG00000128311	*TST*	2	3.65E-02	rs73056793
ENSG00000101290	*CDS2*	2	3.65E-02	rs10041937
ENSG00000138074	*SLC5A6*	2	3.67E-02	rs73055416

ENSG00000110717	*NDUFS8*	1	3.68E-02	rs2078188
ENSG00000181856	*SLC2A4*	2	3.69E-02	rs10941842
ENSG00000130717	*UCK1*	2	3.71E-02	rs10060069
ENSG00000006625	*GGCT*	3	3.72E-02	rs62351320
ENSG00000100033	*PRODH*	2	3.73E-02	rs11952071
ENSG00000119772	*DNMT3A*	2	3.73E-02	rs73055416
ENSG00000106992	*AK1*	2	3.74E-02	rs10060069
ENSG00000114770	*ABCC5*	4	3.82E-02	rs4288117
ENSG00000162551	*ALPL*	2	3.82E-02	rs164448
ENSG00000085871	*MGST2*	2	3.82E-02	rs1911845
ENSG00000243480	*AMY2A*	4	3.85E-02	rs16888249
ENSG00000169239	*CA5B*	2	3.85E-02	rs12652350
ENSG00000135697	*BCO1*	4	3.86E-02	rs3112489
ENSG00000117410	*ATP6V0B*	4	3.90E-02	rs4554223
ENSG00000109390	*NDUFC1*	4	3.91E-02	rs164448
ENSG00000065923	*SLC9A7*	3	3.95E-02	rs4866177
ENSG00000138621	*PPCDC*	2	3.95E-02	rs13357704
ENSG00000059377	*TBXAS1*	2	3.97E-02	rs1911845
ENSG00000151012	*SLC7A11*	2	4.02E-02	rs10069647
ENSG00000103044	*HAS3*	2	4.03E-02	rs4404681
ENSG00000198682	*PAPSS2*	2	4.05E-02	rs2471154
ENSG00000176383	*B3GNT4*	4	4.06E-02	rs13183491
ENSG00000159082	*SYNJ1*	2	4.09E-02	rs4866039
ENSG00000131495	*NDUFA2*	3	4.10E-02	rs6872966
ENSG00000136888	*ATP6V1G1*	4	4.10E-02	rs55822426
ENSG00000104774	*MAN2B1*	2	4.14E-02	rs58017518
ENSG00000107159	*CA9*	2	4.15E-02	rs6887118
ENSG00000167701	*GPT*	2	4.20E-02	rs1521023
ENSG00000241644	*INMT*	2	4.20E-02	rs13153274
ENSG00000184588	*PDE4B*	2	4.22E-02	rs1508547
ENSG00000129167	*TPH1*	2	4.26E-02	rs34294319
ENSG00000115652	*UXS1*	2	4.29E-02	rs11740264
ENSG00000116984	*MTR*	4	4.31E-02	rs10473357
ENSG00000163541	*SUCLG1*	4	4.32E-02	rs55822426
ENSG00000159640	*ACE*	2	4.45E-02	rs4401574
ENSG00000128928	*IVD*	4	4.49E-02	rs71610668
ENSG00000131067	*GGT7*	2	4.49E-02	rs13177416
ENSG00000180817	*PPA1*	3	4.52E-02	rs62351320
ENSG00000185344	*ATP6V0A2*	4	4.53E-02	rs2136125
ENSG00000103415	*HMOX2*	4	4.57E-02	rs4422533
ENSG00000102230	*PCYT1B*	2	4.59E-02	rs986251
ENSG00000156510	*HKDC1*	2	4.64E-02	rs4866039
ENSG00000123739	*PLA2G12A*	2	4.65E-02	rs4866148
ENSG00000169919	*GUSB*	4	4.68E-02	rs7721392
ENSG00000136542	*GALNT5*	2	4.69E-02	rs2136125
ENSG00000101464	*PIGU*	4	4.70E-02	rs67911776
ENSG00000137198	*GMPR*	2	4.73E-02	rs4422533
ENSG00000138604	*GLCE*	2	4.83E-02	rs1508547
ENSG00000065989	*PDE4A*	2	4.86E-02	rs62351320
ENSG00000110921	*MVK*	2	4.90E-02	rs11738280
ENSG00000065357	*DGKA*	4	4.94E-02	rs6451573
ENSG00000104723	*TUSC3*	2	4.97E-02	rs1540751
ENSG00000109323	*MANBA*	4	4.99E-02	rs7734813
ENSG00000145494	*NDUFS6*	4	5.05E-02	rs12110309
ENSG00000187097	*ENTPD5*	2	5.12E-02	rs71610668
ENSG00000141401	*IMPA2*	2	5.18E-02	rs7734813
ENSG00000196177	*ACADSB*	4	5.19E-02	rs7734813
ENSG00000006757	*PNPLA4*	2	5.23E-02	rs12110309

ENSG00000116761	*CTH*	2	5.28E-02	rs7734813
ENSG00000151689	*INPP1*	2	5.29E-02	rs10045245
ENSG00000079462	*PAFAH1B3*	1	5.29E-02	rs7720726
ENSG00000151151	*IPMK*	4	5.31E-02	rs11738280
ENSG00000169255	*B3GALNT1*	2	5.35E-02	rs10065518
ENSG00000241973	*PI4KA*	2	5.42E-02	rs4404681
ENSG00000139163	*ETNK1*	4	5.43E-02	rs1909117
ENSG00000166507	*NDST2*	2	5.46E-02	rs71610668
ENSG00000100422	*CERK*	4	5.54E-02	rs7734813
ENSG00000011405	*PIK3C2A*	4	5.65E-02	rs71610668
ENSG00000135940	*COX5B*	1	5.71E-02	rs9292895
ENSG00000172269	*DPAGT1*	4	5.73E-02	rs1508547
ENSG00000131100	*ATP6V1E1*	4	5.80E-02	rs11744487
ENSG00000186010	*NDUFA13*	3	5.87E-02	rs919344
ENSG00000214617	*SLC6A10P*	4	5.95E-02	rs4554223
ENSG00000182621	*PLCB1*	2	5.98E-02	rs2202798
ENSG00000173262	*SLC2A14*	4	6.00E-02	rs12110309
ENSG00000138030	*KHK*	4	6.01E-02	rs7734813
ENSG00000129562	*DAD1*	4	6.11E-02	rs10079950
ENSG00000074410	*CA12*	2	6.19E-02	rs1521026
ENSG00000108381	*ASPA*	2	6.28E-02	rs10805661
ENSG00000162104	*ADCY9*	2	6.29E-02	rs35773719
ENSG00000010404	*IDS*	4	6.82E-02	rs6887953
ENSG00000124713	*GNMT*	2	6.98E-02	rs1508547
ENSG00000188641	*DPYD*	2	7.42E-02	rs1508547
ENSG00000182224	*CYB5D1*	4	7.52E-02	rs4866150
ENSG00000140199	*SLC12A6*	2	7.58E-02	rs1703045
ENSG00000133475	*GGT2*	4	7.58E-02	rs7720726
ENSG00000170906	*NDUFA3*	4	7.96E-02	rs6872966
ENSG00000133063	*CHIT1*	2	8.00E-02	rs1435983
ENSG00000135677	*GNS*	4	8.84E-02	rs55749186
ENSG00000079215	*SLC1A3*	2	9.27E-02	rs10069647
ENSG00000112874	*NUDT12*	2	1.09E-01	rs13190608
